# Identification and validation of urinary CXCL9 as a biomarker for diagnosis of acute interstitial nephritis

**DOI:** 10.1172/JCI168950

**Published:** 2023-07-03

**Authors:** Dennis G. Moledina, Wassim Obeid, Rex N. Smith, Ivy Rosales, Meghan E. Sise, Gilbert Moeckel, Michael Kashgarian, Michael Kuperman, Kirk N. Campbell, Sean Lefferts, Kristin Meliambro, Markus Bitzer, Mark A. Perazella, Randy L. Luciano, Jordan S. Pober, Lloyd G. Cantley, Robert B. Colvin, F. Perry Wilson, Chirag R. Parikh

**Affiliations:** 1 Section of Nephrology, Department of Internal Medicine and; 2 Clinical and Translational Research Accelerator, Department of Internal Medicine, Yale School of Medicine, New Haven, Connecticut, USA.; 3 Division of Nephrology, Internal Medicine, Johns Hopkins School of Medicine, Baltimore, Maryland, USA.; 4 Department of Pathology, Massachusetts General Hospital, Boston, Massachusetts, USA.; 5 Department of Pathology, Harvard Medical School, Boston, Massachusetts, USA.; 6 Immunopathology Research Laboratory and; 7 Section of Nephrology, Department of Internal Medicine, Massachusetts General Hospital, Boston, Massachusetts, USA.; 8 Section of Renal Pathology, Department of Pathology, Yale School of Medicine, New Haven, Connecticut, USA.; 9 Arkana Labs, Little Rock, Arkansas, USA.; 10 Division of Nephrology, Department of Internal Medicine, Icahn School of Medicine at Mount Sinai, New York, New York, USA.; 11 Section of Nephrology, Department of Internal Medicine, University of Michigan, Ann Arbor, Michigan, USA.; 12 Department of Pathology and; 13 Department of Immunobiology, Yale School of Medicine, New Haven, Connecticut, USA.

**Keywords:** Nephrology, Chemokines, Chronic kidney disease, Diagnostics

## Abstract

**Background:**

Acute tubulointerstitial nephritis (AIN) is one of the few causes of acute kidney injury with diagnosis-specific treatment options. However, due to the need to obtain a kidney biopsy for histological confirmation, AIN diagnosis can be delayed, missed, or incorrectly assumed. Here, we identify and validate urinary CXCL9, an IFN-γ-induced chemokine involved in lymphocyte chemotaxis, as a diagnostic biomarker for AIN.

**Methods:**

In a prospectively enrolled cohort with pathologist-adjudicated histological diagnoses, termed the discovery cohort, we tested the association of 180 immune proteins measured by a proximity extension assay with AIN and validated the top protein, CXCL9, using sandwich immunoassay. We externally validated these findings in 2 cohorts with biopsy-confirmed diagnoses, termed the validation cohorts, and examined mRNA expression differences in kidney tissue from patients with AIN and individuals in the control group.

**Results:**

In a proximity extension assay, urinary CXCL9 was 7.6-fold higher in patients with AIN than in individuals in the control group (*P* = 1.23 × 10^–5^). Urinary CXCL9 measured by sandwich immunoassay was associated with AIN in the discovery cohort (*n* = 204; 15% AIN) independently of currently available clinical tests for AIN (adjusted odds ratio for highest versus lowest quartile: 6.0 [1.8–20]). Similar findings were noted in external validation cohorts, where CXCL9 had an AUC of 0.94 (0.86–1.00) for AIN diagnosis. *CXCL9* mRNA expression was 3.9-fold higher in kidney tissue from patients with AIN (*n* = 19) compared with individuals in the control group (*n* = 52; *P* = 5.8 × 10^–6^).

**Conclusion:**

We identified CXCL9 as a diagnostic biomarker for AIN using proximity extension urine proteomics, confirmed this association using sandwich immunoassays in discovery and external validation cohorts, and observed higher expression of this protein in kidney biopsies from patients with AIN.

**Funding:**

This study was supported by National Institute of Diabetes and Digestive and Kidney Diseases (NIDDK) awards K23DK117065 (DGM), K08DK113281 (KM), R01DK128087 (DGM), R01DK126815 (DGM and LGC), R01DK126477 (KNC), UH3DK114866 (CRP, DGM, and FPW), R01DK130839 (MES), and P30DK079310 (the Yale O’Brien Center). The content is solely the responsibility of the authors and does not necessarily represent the official views of the National Institutes of Health.

## Graphical abstract

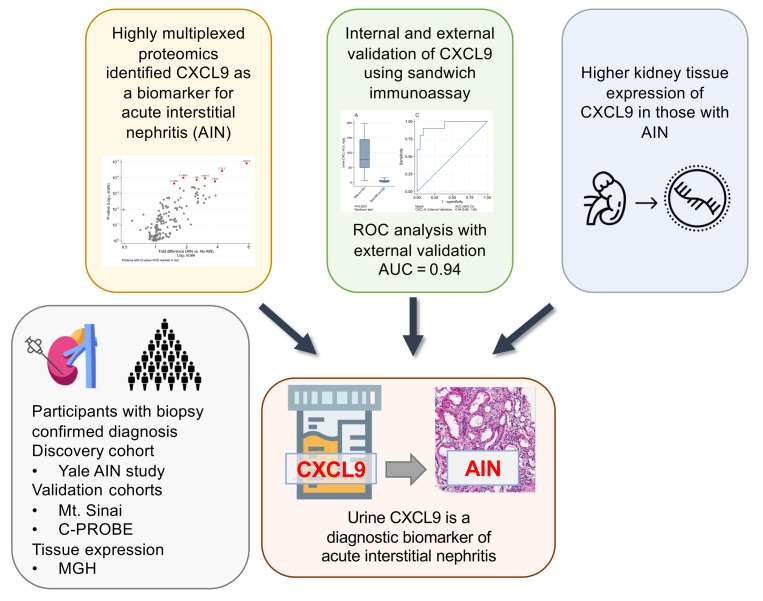

## Introduction

Sudden loss of kidney function, termed acute kidney injury (AKI), is common, affecting 1 in 5 hospitalized patients and is associated with adverse long-term outcomes, including chronic kidney disease, end-stage kidney disease, and death (1–6). Clinically, many underlying injuries can present as AKI, all of which require a unique management strategy; for example, acute tubular injury (ATI) has no diagnosis-specific treatment and requires watchful waiting, whereas acute tubulointerstitial nephritis (AIN) can be treated by withdrawal of a culprit drug or immunosuppressive therapy (7). Therefore, evaluation of a patient with AKI requires a thorough evaluation of the underlying cause of AKI and development of a personalized treatment plan focused on the underlying etiology and mechanism of injury.

A major challenge in the clinical care of patients with AKI is differentiating AIN from other causes of AKI (8, 9). Most patients with AIN have no disease-specific signs or symptoms (10). Clinically available tests, such as urine eosinophils, urine microscopy for WBC casts, and imaging tests have poor accuracy (11–14). Therefore, diagnosis of AIN often requires a kidney biopsy, which adds procedure-related risks (15, 16). It also leads to a delay in care that is associated with lower chances of kidney function recovery (17). In fact, about half of patients suffer significant, permanent kidney damage after an episode of AIN, which may be compounded, in part, by a delay in diagnosis (10). Thus, identification of an accurate, noninvasive diagnostic biomarker for AIN is likely to improve the care of patients with AKI who are suspected to have AIN.

In this study, we sought to discover and validate novel biomarkers for diagnosing AIN in a cohort of prospectively enrolled participants with biopsy-confirmed and pathologist-adjudicated AIN and a control group. Previously, in hypothesis-driven targeted testing, we showed that 2 cytokines in the urine, TNF-α and IL-9, differentiated between patients with AIN and individuals in the control group with greater accuracy than both clinicians’ prebiopsy suspicion for AIN and a model of currently available clinical tests for AIN (18, 19). Here, we performed urine proteomics to identify additional candidate biomarkers that can improve diagnostic accuracy, then confirmed the proteomics findings by using sandwich immunoassays to measure the top candidate biomarker, chemokine C-X-C motif ligand 9 (CXCL9), in the discovery cohort, externally validated the biomarker findings, and demonstrated higher expression of CXCL9 in kidney tissue from patients with AIN than in individuals in the control group.

## Results

### Urine proteomics analysis identified CXCL9 as the top protein biomarker distinguishing AIN from other causes of AKI.

**Figure 1:**
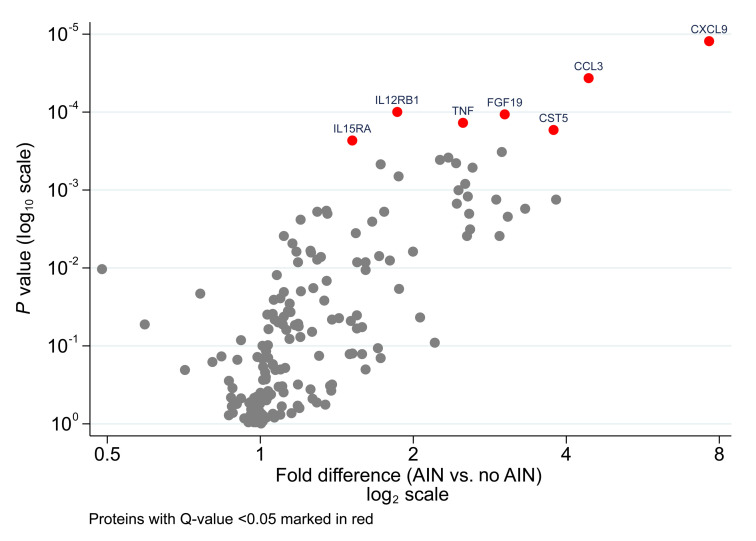
Volcano plot demonstrating associations of proximity extension measurement of urine proteins with acute interstitial nephritis diagnosis. Proteins with Q values of less than 0.05 using the Benjamini-Hochberg procedure are highlighted in red.

We included 88 participants in our urine proteomic analysis, which included 31 (35%) participants with biopsy-confirmed, pathologist-adjudicated AIN and 57 (65%) with a random spectrum of other diagnoses as controls (Supplemental Table 1; supplemental material available online with this article; https://doi.org/10.1172/JCI168950DS1). These participants had comparable characteristics at biopsy, except that patients with AIN tended to have higher serum creatinine levels and lower urine albumin-to-creatinine ratios (Supplemental Table 2). Of 180 proteins evaluated, 7 had FDRs (Benjamini Hochberg *Q* value) and Bonferroni corrected *P* values of under 0.05 (Supplemental Table 3). Among these, CXCL9 had the greatest strength of association (*P* = 1.23 × 10^–5^) and was 7.6-fold higher in participants with AIN compared with individuals in the control group (Figure 1). We also noted that TNF-α was 2.5-fold higher in participants with AIN than in individuals in the control group (*P* = 1.37 × 10^–4^), which we previously identified using a targeted approach (18). Other IFN-γ induced chemokines, CXCL10 and CXCL11, were comparable between patients and individuals in the control group (Supplemental Table 3). Similar results were noted when indexing urine protein values to urine creatinine (Supplemental Figure 1) or when only including proteins detected in over 75% of samples (Supplemental Figure 2). Pathway analysis suggested that the top upstream regulators of the observed changes were the proinflammatory cytokine IFN-γ (predicted state: activated), which is the key upstream regulator of CXCL9, and IL-10 (predicted state: inhibited), which is known to suppress inflammation (Supplemental Table 4).

### Urinary CXCL9 was higher with AIN diagnosis and with higher severity of histological markers of AIN severity.

We used a modified sandwich immunoassay to measure CXCL9 in a cohort of 204 consecutive participants at 2 Yale-affiliated hospitals who underwent kidney biopsy for evaluation of acute kidney disease (Supplemental Figure 3). Among these participants, 31 (15%) were adjudicated as having AIN by all 3 study pathologists. We noted a high correlation between CXCL9 measured by urine proteomics and by immunoassay (correlation coefficient = 0.99) (Supplemental Figure 4). Participants with higher CXCL9 tended to be older, had higher urine albumin-to-creatinine ratios and higher serum creatinine at biopsy, and were more likely to have AKI and AKI that requires dialysis (Table 1). Those with higher CXCL9 were more likely to have been prescribed immune-checkpoint inhibitors and have greater occurrence of leukocytes on urine microscopy and dipstick analysis.

**Figure 2:**
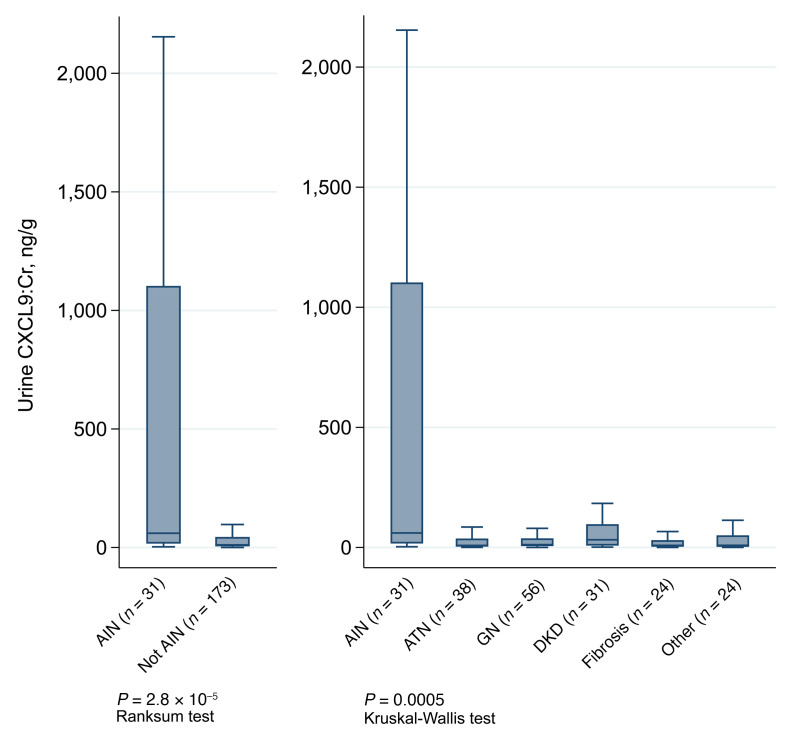
Urine CXCL9 levels are higher in acute interstitial nephritis compared with controls in the discovery cohort. Box and whisker plots of CXCL9 by presence or absence of AIN (left panel) and by histological diagnosis in the discovery cohort (right panel). Boxes represent interquartile range and horizontal line within box represents median. Median and interquartile range values are presented in Table 2.

CXCL9 levels were 5.5-fold higher in those with AIN than in individuals in the control group and between AIN and various other kidney diseases (Figure 2). Similar results were noted when we used CXCL9 values without indexing to urine creatinine (Supplemental Figure 5). The association of CXCL9 with AIN was consistent, regardless of the criteria used to define AIN (Table 2). Median CXCL9 levels were 8-fold higher in those with AIN when comparing to those with ATI (AIN versus ATI, 60.3 [16.4, 1103.4] versus 7.7 [3.3, 28.7]; *P* = 0.0001; Supplemental Figure 6). CXCL9 was associated with severity of interstitial features characteristic of AIN, including interstitial infiltrate and tubulitis, but not with the degree of interstitial eosinophilia or tubular injury (Supplemental Figure 7). CXCL9 was not associated with glomerular crescents, a marker of glomerular inflammatory damage, which is not a typical feature of AIN (Supplemental Figure 8).

### Urinary CXCL9 was independently associated with AIN diagnosis and improved the AUC for AIN diagnosis over currently available clinical information.

**Figure 3:**
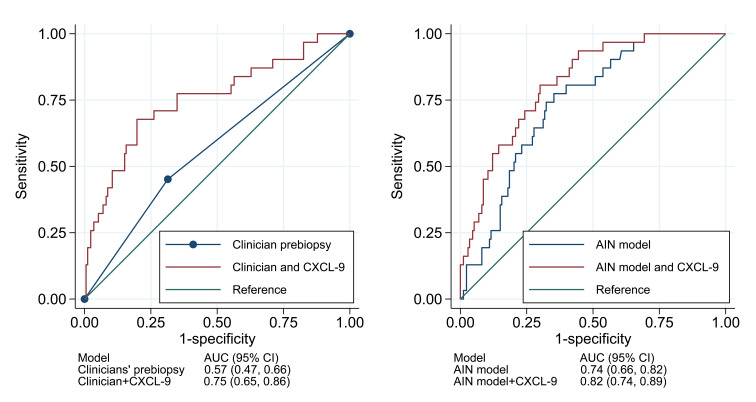
Urine CXCL9 improved the AUC for acute interstitial nephritis compared with existing information. Comparison of AUC of CXCL9 for AIN diagnosis compared with clinicians’ prebiopsy diagnosis of acute interstitial nephritis obtained through chart review (left panel) and AIN statistical model as described in ref. 19 (right panel)

We noted 40% higher odds of AIN per doubling of CXCL9 (OR: 1.4; 95% CI: 1.2–1.5). Compared with those with CXCL9 values in the lowest quartile, those in the highest quartile had 6-fold higher odds of AIN (OR: 6.0; 95% CI: 1.8–19.9) (Table 3, Model 1). We noted similar results in multivariable analyses controlling for clinicians’ prebiopsy suspicion for AIN (Table 3, Model 2) and for an externally validated statistical model for AIN (Table 3, Model 3). The association of urinary CXCL9 with AIN was independent of demographics, comorbidities, plasma CXCL9, and 2 previously described urine biomarkers of AIN, IL-9 and TNF-α (Supplemental Table 5). Addition of CXCL9 improved the AUC over clinicians’ prebiopsy impression by 0.18 to 0.75 (95% CI: 0.65–0.86) and over the AIN diagnostic model by 0.08–0.82 (95% CI: 0.74–0.89) (Figure 3). The AUC of CXCL9 for differentiating AIN from ATI was 0.77 (0.66, 0.88) (Supplemental Figure 6).

### External validation cohorts.

**Figure 4:**
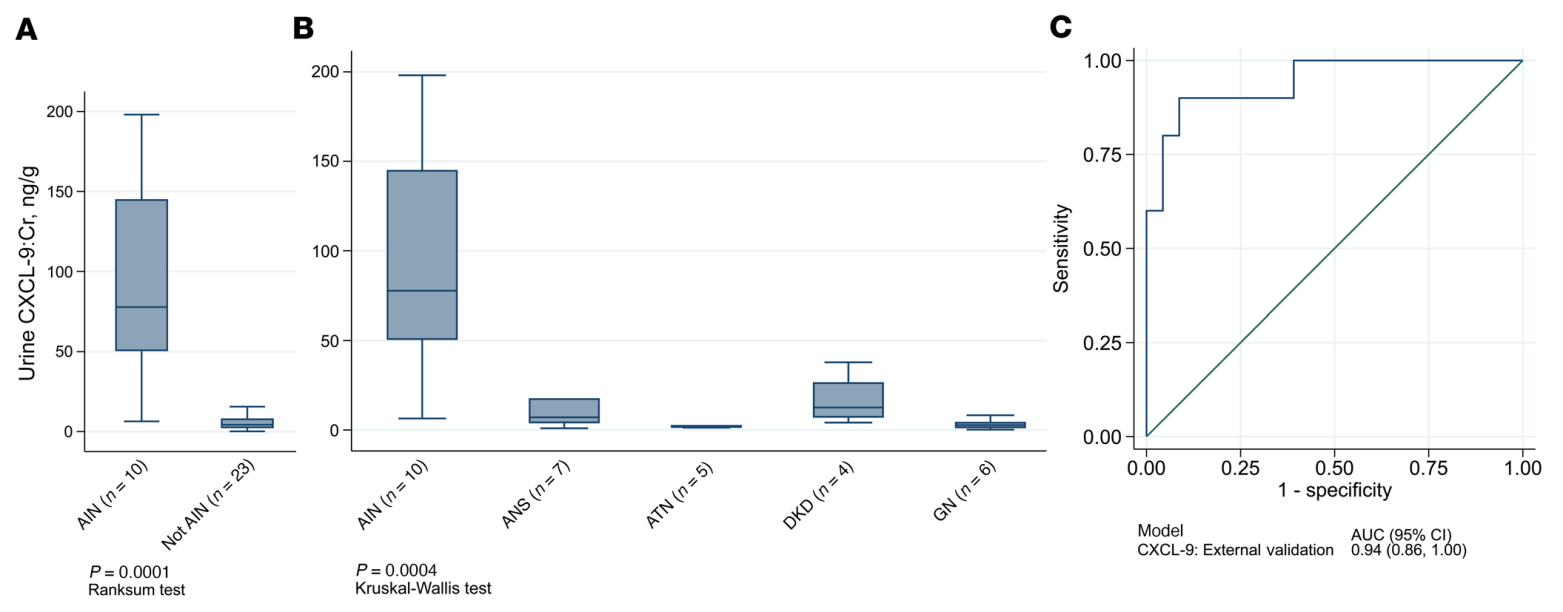
CXCL9 levels were higher in AIN than in controls in the external validation cohorts. Box plot of CXCL9 by presence or absence of AIN (**A**) and by histological diagnosis in the validation cohort (**B**). (**C**) AUC of CXCL9 for AIN diagnosis. AIN, acute interstitial nephritis; ANS, arterionephrosclerosis; ATN, acute tubular necrosis/injury; DKD, diabetic kidney disease; GN, glomerulonephritis

We validated our findings in 2 external cohorts with histologically confirmed diagnoses: C-PROBE (*n* = 12; AIN = 4) and Icahn School of Medicine (*n* = 21; AIN = 6). Participants’ characteristics are presented in Supplemental Table 6. We noted that CXCL9 levels were higher in patients with AIN than in the individuals in the non-AIN control group (Figure 4, A and B). We noted that CXCL9 levels were higher in patients with AIN than in the individuals in the non-AIN control group in each external validation cohort (Supplemental Figure 9), and there was no difference in the association of CXCL9 with AIN diagnosis by site (interaction *P* = 0.83). CXCL9 had an AUC of 0.94 (95% CI: 0.86–1.00) for AIN diagnosis in the external validation cohorts (Figure 4C).

### Urinary CXCL9 test characteristics for AIN diagnosis.

We present test characteristics of urinary CXCL9 in the discovery and external validation cohorts at 4 cutpoints derived from the discovery cohort: the 25th, 50th, and 75th percentiles, and a cutpoint derived by maximizing the sum of sensitivity and specificity using the Youden index (Table 4). At a cutpoint corresponding to the median value from the discovery cohort (14.2 ng/g), CXCL9 had sensitivities of 81% and 90% in the discovery and validation cohorts, respectively, and negative predictive values of 94% and 95%, respectively. At the 75th percentile cutpoint (58.9 ng/g), we noted specificities of 79% and 100% and positive predictive values of 30% and 100%, respectively, in the discovery and validation cohorts.

### Patients with AIN had higher kidney tissue mRNA expression of CXCL9 than individuals in the non-AIN control group.

**Figure 5:**
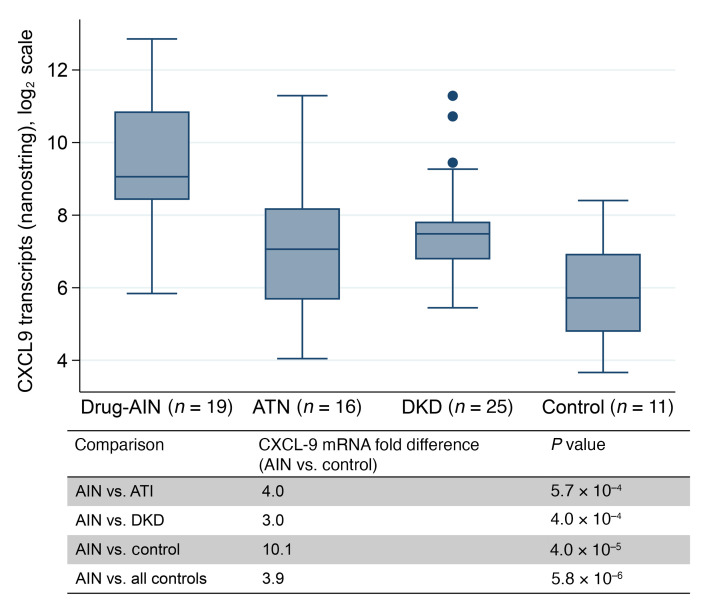
CXCL9 expression in kidney biopsies was higher in acute interstitial nephritis than in controls. All native kidneys except ATI (transplant); Control, histologically normal biopsies; Nanostring analysis; Kruskal Wallis or Wilcoxon Rank-sum test

We compared kidney tissue expression of CXCL9 in patients with AIN (*n* = 19) versus those with other diagnoses (*n* = 52) using Nanostring analysis. We noted higher tissue mRNA expression of CXCL9 in biopsies from patients with AIN than in biopsies from patients with diabetic kidney disease, ATI, and individuals in the control group (Figure 5). Among the top proteins that differed between patients with AIN versus individuals in the control group, we noted that a majority are known to be induced by IFN-γ (Supplemental Table 7).

### Association of CXCL10 with AIN.

Similar to CXCL9, CXCL10 is also induced by IFN-γ and binds to their shared receptor, CXCR3. We noted a moderately high correlation coefficient between the 2 chemokines (ρ=0.71, *P* < 0.001). CXCL10 levels were twice as high in patients with AIN than in individuals in the control group (117 [40.2, 845] versus 60.0 [26.8, 170]) and the odds of AIN were 1.3-fold higher per doubling in urine CXCL10 (OR: 1.31 [1.12, 1.53]). However, when the analysis was controlled for CXCL9, the association of CXCL10 with AIN was no longer significant (adjusted OR, 0.95 [0.75, 1.22]), whereas the association of CXCL9 with AIN was independent of CXCL10 levels (Supplemental Table 5).

### Biomarker combinations.

To determine the optimal biomarker combination for AIN diagnosis, we used the LASSO feature selection algorithm to determine the optimal combination of biomarkers among all biomarkers measured in our cohort. In 1,000 iterations of 70% random subset selection of the cohort, we noted that IL-9, TNF-α, and CXCL9 were selected in over 75% of models (Supplemental Figure 10). We trained a logistic model for these 3 biomarkers for the outcome of AIN on the first 70% of discovery cohort participants (training set) and applied these model weights to the next 30% of discovery cohort participants (test set) and the external validation cohorts. We noted an AUC of 0.89 (95% CI: 0.77–0.98) in the test set and 0.87 (95% CI: 0.70–0.99) in the external validation cohort (Supplemental Figure 11). Precision recall curves and calibration plots are presented in Supplemental Figures 12 and 13. At a model probability cutoff of 10%, we noted sensitivity and specificity of 87% and 60%, respectively, in the test set and 90% and 70%, respectively, in the external validation set for the model containing all 3 biomarkers (Supplemental Table 8).

## Discussion

In a cohort of participants including patients with biopsy-confirmed AIN and individuals in the non-AIN control group, we identified and validated CXCL9 as a diagnostic biomarker for AIN. In proteomic analysis, among the 180 proteins in the urine, CXCL9 showed the best diagnostic accuracy for AIN. We confirmed the association of urinary CXCL9 with AIN using sandwich immunoassays in the discovery and external validation cohorts. CXCL9 improved the diagnostic accuracy of AIN over clinicians’ prebiopsy suspicion for AIN, a validated statistical model for AIN diagnosis using currently available clinical tests, and 2 previously identified biomarkers of AIN, TNF-α and IL-9. We also show higher expression of CXCL9 in kidney tissue from patients with AIN. Finally, we showed that urinary CXCL9 together with TNF-α and IL-9 is the optimal combination of biomarkers for AIN diagnosis.

CXCL9, also known as monokine induced by IFN-γ, is a chemokine that binds to its receptor, CXCR-3, and promotes lymphocyte recruitment at sites of inflammation. CXCL9 has been shown to have a role in promoting kidney tubulointerstitial inflammation. One study showed lower levels of interstitial infiltrate in CXCL9 knockout mice (20). CXCL9 is also associated with acute kidney allograft rejection (21), future risk of rejection (22), and subclinical rejection (23). Allograft rejection has significant tissue transcriptomic overlap with AIN, including similar mRNA expression of CXCL9 (24), which could explain the association of CXCL9 with both transplant rejection and AIN. In a recent paper, Singh, et al. noted higher CXCL9 expression in kidney biopsies from patients with immune checkpoint inhibitor therapy–associated AIN (25). In another recent study, Nunez et al. showed that a rise in plasma CXCL9 occurring 1–2 weeks after starting immune checkpoint–inhibitor therapy predicted future occurrence of immune-related adverse events, including AIN (26). CXCL9 also appears to be a marker of inflammation restricted to the tubulointerstitial, rather than the glomerular, space. For example, Schmidt et al. reported that plasma CXCL9 was one of the strongest predictors of interstitial (but not glomerular) inflammation in the Boston Kidney Biopsy Cohort (27). We similarly noted that CXCL9 was associated with findings of tubulointerstitial inflammation such as tubulitis and interstitial infiltrate, but not with glomerular crescents. Another recent study showed that CXCL10, a related IFN-γ–induced chemokine, was higher in patients with AIN than in individuals in the control group (28).Our proteomics analysis did not show a significant difference in CXCL10 levels between patients with AIN and individuals in the non-AIN control group. Moreover, CXCL9 was significantly associated with AIN after controlling for CXCL10, but not vice versa. CXCL9, CXCL10, and related chemokine CXCL11 are induced in macrophages by IFN-γ and bind to the same chemokine receptor, namely, CXCR3. However, different cell types may preferentially express different ratios of these chemokines. We do not know which cell type is the predominant source of CXCL9 in AIN.

AIN is one of the few causes of AKI that has a specific treatment, and timely confirmation of the diagnosis could lead to disease-specific management strategies, such as withdrawal of the culprit drug and administration of corticosteroid therapy. However, due to the need for and risks associated with kidney biopsy, AIN diagnosis is often delayed, resulting in permanent kidney damage. In some cases of suspected AIN, kidney biopsy cannot be safely performed in a timely manner due to use of antiplatelet and anticoagulant medications. Therefore, clinicians often assume the diagnosis in an effort to avoid kidney biopsy risks and instead try withdrawal of all potential culprit drugs and administration of corticosteroid therapy (29). This approach can lead to harms of overtreatment if the assumption of AIN diagnosis is incorrect; discontinued medications may include life-saving therapies such as antibiotics and anticancer medications, and corticosteroid therapy carries risks such as hyperglycemia, bone loss, gastrointestinal hemorrhage, and infection. Our data show that, in patients with suspected AIN, the urine biomarker CXCL9 can significantly improve clinical care by helping to rule in or rule out the disease in a large subset of patients, and kidney biopsy can be reserved for a narrower subset in whom biomarker values are equivocal. For example, urinary CXCL9:creatinine value below 14.2 ng/g could be used to rule out AIN with negative predictive values exceeding 94% and avoid the need to withdraw lifesaving medications or administer corticosteroids. Urinary CXCL9:creatinine values above 58.9 ng/g could be used to rule in AIN with initiation of empiric therapy, whereas those with CXCL9:creatinine values between 14.2 and 58.9 may still need a biopsy to confirm the diagnosis.

Our findings can guide future research into diagnosis and therapy. First, medical therapy for AIN is limited to use of corticosteroids that provide broad immunosuppression with many adverse effects. Despite therapy, many patients with AIN develop permanent kidney damage and chronic kidney disease. We used both urine proteomics and tissue transcriptomics to identify IFN-γ as a key upstream regulator of the inflammatory changes observed in AIN. This should lead to investigation of therapies targeting IFN-γ for treatment of AIN. Second, we noted that CXCL9 values were over 100-fold higher than IL-9 and TNF-α values, consistent with the higher concentrations of chemokines, compared with cytokines, required to engage relevant receptors. Importantly, the greater concentration of CXCL9 portends improved laboratory test characteristics, and CXCL9 has the potential to be included in existing platforms in clinical laboratories or developed as a point-of-care test. In fact, point-of-care devices identifying CXCL9 are currently under development for diagnosis of acute cell-mediated transplant rejection, which, similar to AIN, is a tubulointerstitial immune-mediated process that spares the glomeruli, and such devices could be adapted for rapid, bedside diagnosis of AIN (30). Our findings also provide insight into AIN pathogenesis. In contrast with the marked upregulation of CXCL9 in tissue and urine from patients with AIN, patients with ATI had very low levels of CXCL9. Consistent with this, our recent analysis of the cellular events underlying ATI in murine models of ischemia-reperfusion injury demonstrated almost no upregulation of *Cxcl9* in the kidney, despite T cell recruitment to the interstitial compartment (31). These data suggest that CXCL9 might not only serve as a clinical biomarker to distinguish AIN from ATI, but that it might also be a key biological regulator of the T cell activation states that promote destructive responses, such as tubulitis, that are commonly seen in AIN and rarely seen in ATI. We did not examine why CXCL9 shows a strong association with interstitial inflammation, but similar association is not noted for its related IFN-γ–induced chemokines CXCL-10 or -11. One potential explanation could be that there are different cellular targets of IFN-γ, which lead to preferential expression of one chemokine over another, as seen in other organs (32). This could be answered by future studies using techniques such as in situ hybridization or single cell RNA-Seq of kidney biopsy tissue.

Strengths of our study include use of biopsy-confirmed, pathologist-adjudicated patients with AIN and a control group and prospective collection of samples and data. Another strength of our study is the validation of proteomics findings using immunoassay and tissue expression, as well as the inclusion of external validation cohorts. Our study also has some limitations. First, we compared CXCL9 levels to pathologist-defined AIN diagnosis. However, several studies show poor inter-rater agreement for renal histological diagnosis and features (18, 33). To overcome this limitation, we compared CXCL9 levels with various alternative diagnoses. We believe that this additional uncertainty in diagnosis likely imposed an upper limit on the observed accuracy, and a more certain diagnosis of AIN could have shown even greater accuracy. Second, due to the limited sample size, we could not compare biomarker levels between the various etiologies of AIN (e.g., drug versus autoimmune AIN and AIN due to different drug classes), which needs to be explored in a future, larger study. Moreover, in this study, we pursued the top proteomic hit, CXCL9; however, it is possible that other proteins in the proteomics panel might provide additional, potentially orthogonal information. For example, CCL3, a major eosinophilic attractant, was also significantly higher in patients with AIN than in the control group and might serve to identify patients with AIN due to antibiotics, which tend to have a higher number of interstitial eosinophils. We hope to pursue such phenotyping efforts in a future study. Third, we did not collect samples longitudinally to assess temporal changes in CXCL9 levels, as patients with AIN receive treatment. Fourth, while the study provides preliminary insights into the pathways that might be dysregulated in AIN (e.g., IFN-γ), detailed mechanistic analyses were beyond the scope of our current study. Finally, it is possible that urinary CXCL9 may have originated in other organs, entered the systemic circulation, and was then filtered out into the urine; however, the higher expression of CXCL9 mRNA in kidney biopsies from patients with AIN suggest that the urinary CXCL9 likely originated in the kidneys. Similarly, higher expression of CXCL9 in biopsies from patients with immune checkpoint inhibitor–related AIN was noted by Singh, et al. (25).

In conclusion, we identified CXCL9 as a biomarker of AIN using urine proteomics with confirmation by sandwich immunoassays, external validation, and tissue expression. We demonstrated the independent association of CXCL9 with AIN diagnosis. The future development of clinically useful assays for detection of these biomarkers in urine samples may prospectively assess the utility of biomarker information for prognostication of clinical outcomes.

## Methods

### Study design and participants.

The discovery cohort included participants enrolled in the Yale biopsy cohort, which has been previously described (18, 34). Briefly, we prospectively enrolled patients undergoing a kidney biopsy at 2 Yale-affiliated sites, Yale New Haven Hospital and Saint Raphael’s Hospital, between January 2015 and June 2018. Both hospitals are in New Haven, Connecticut, USA. We enrolled participants using consecutive sampling, excluding patients undergoing biopsies for evaluation of transplanted kidneys or kidney malignancies. For this substudy, we excluded participants who either failed to provide a urine sample for analysis, did not undergo a biopsy after enrollment, had insufficient tissue for histological diagnosis, or underwent a biopsy for indications other than AKI or acute kidney disease, which were defined using the Kidney Diseases: Improving Global outcomes (KDIGO) serum creatinine criteria (35).

### Outcome: histological AIN diagnosis.

The primary outcome in the discovery cohort was histological, pathologist-adjudicated AIN diagnosis. Three renal pathologists evaluated biopsy slides from all study participants with an official biopsy report of AIN and a subset of those with biopsy reports of other diagnoses. The pathologists reported the presence or absence of an AIN diagnosis on histological analysis independent of each other and were blinded to the clinical history and official biopsy report. Inter-rater agreement for AIN diagnosis was 63%–70% with a Fleiss k of 0.35 (18). We defined a participant as an AIN case when all 3 pathologists classified their biopsy as AIN. We defined a participant as a not-AIN control when none reported AIN. In our primary analysis, we excluded biopsies without consensus on AIN status but included them in a sensitivity analysis where we ascertained case or control status based on reports from a majority of pathologists. In 2 additional sensitivity analyses, we defined cases and controls based on the diagnoses of the treating nephrologists after their review of the biopsies and based on the official biopsy interpretation. We also collected information on interstitial histological features through adjudication and review of biopsy reports.

### Urine proteomics.

Urine samples were collected a median of 6.2 (IQR: 1.6–26.7) hours before the biopsy, and urine supernatants were stored at –80°C. We performed proteomics and biomarker measurements from urine samples after a single controlled thaw. The sample processing protocol and biorepository tracking details were described in a prior publication from our group (36). In this analysis, we included all participants in the discovery cohort adjudicated as AIN by all 3 pathologists and a random subset of controls with histological diagnoses other than AIN. Urine proteomic measurements were performed by Olink Proteomics using 2 commercially available, manufacturer-validated panels named immune response (v.3203) and inflammation (v.3021). Of the 184 proteins included across the 2 panels, we included 180 that were nonoverlapping (Supplemental Table 9). The Olink Proximity Extension Assay (PEA) is a high-throughput, multiplexed, proteomic platform. Two PEA probes (oligonucleotide-labeled monoclonal or polyclonal antibodies) separately bind each target protein to minimize cross-reactivity. Upon binding, the complementary probes for each target hybridize and extend, generating a unique sequence used for digital identification of each specific protein. Sequencing was performed on a NovaSeq 6000 system (Illumina). The amounts of known sequences are translated into Normalized Protein eXpression (NPX) units on a log_2_ scale derived from count (Ct) values. Quality-control data from Olink analyses are presented in Supplemental Table 10. Olink performed measurements blinded to case status and provided results to investigators. Olink had no role in statistical analysis or publication.

### Urinary CXCL9 using sandwich immunoassay.

In participants from both the discovery and the external validation cohorts, we analyzed urine samples using the CXCL9 R-plex assay on the Mesoscale discovery platform (Meso Scale Diagnostics). The Mesoscale discovery platform is a modified sandwich immunoassay that uses electrochemiluminescence to determine protein concentrations. The assay was developed and validated in house, with an average dilution-linearity percent recovery of 101% (92.5%–109%) and average spike recovery of 89% (83%–95%). The dynamic range of the assay is 0.24–8,000 pg/mL. Inter- and intra-assay coefficients of variation were 11% and 2%, respectively, and 99% of values were within the detection range (Supplemental Table 11). We normalized all urine biomarkers to urine creatinine to account for urine concentration differences. We performed urine albumin and creatinine measurements using a Randox RX Daytona machine and urine dipstick analysis using a Clinitek Status analyzer (Siemens Healthcare Diagnostics Inc.). We also performed urine sediment microscopy and took representative pictures. Personnel measuring biomarkers and performing urine dipstick analysis and urinalysis were blinded to case status. For comparison, we also included biomarkers previously measured in this cohort and described in our prior publication (18).

### Sources of clinical data.

We collected demographics, clinical histories, laboratory results, medications, and nephrologists’ pre- and postbiopsy diagnoses through chart review of the Epic electronic health record (EHR) (Epic, Inc.) and crossreferenced these data with patient interviews, as previously described (18). We checked scanned laboratory records or called physician’s offices if these data were not available from the EHR. We also reviewed biopsy reports for histological diagnoses and severity of interstitial features.

### Nanostring mRNA assay and analysis.

We analyzed formalin-fixed, paraffin-embedded (FFPE) biopsy blocks from archived samples at Massachusetts General Hospital. All biopsies were obtained as part of routine care and had sufficient remaining tissue after completion of diagnostic studies. Five or 6 consecutive 20 μm curls cut from each FFPE block of kidney tissue were immediately transferred to sterile microcentrifuge tubes and stored at room temperature. We performed deparaffinization and RNA extraction using Quick-RNA FFPE Minipreps (Zymo Research). We measured RNA concentration and purity with a Nano-Drop 2000 spectrophotometer (Thermo Fisher Scientific). We quantified gene expression of the FFPE tissue-derived RNA isolates using the nCounter MAX System (NanoString Technologies). We selected the Banff Human Organ Transplant (B-HOT) 770-gene panel for hybridization (NanoString Technologies) (37) because of the pathologic similarities between AIN and acute cellular rejection and because it is enriched for immune cell genes. Quality control assessment and normalization were performed as previously described (38).

### Validation cohorts.

The validation set included 2 cohorts of participants: the Icahn School of Medicine and the Clinical Phenotyping and Resource Biobank Core (C-PROBE) of the George M. O’Brien Kidney Translational Core Center at the University of Michigan. We selected all participants with AIN who had available urine samples and included 2 participants without AIN per 1 selected AIN participant. At Mount Sinai participants were enrolled between 2014 and 2020. In C-PROBE, participants were enrolled between 2009 and 2016. Full details of these cohorts were previously published (39, 40). For validation cohorts, we defined the presence or absence of AIN based on official biopsy interpretations.

### Statistics.

For urine proteomics analysis, we presented characteristics of participants as median (IQR) or count (percentage) by presence or absence of AIN. We presented Olink urine proteomic results as volcano plots, where we plotted *P* values on the y-axis (log_10_ scale) and fold-difference in biomarker levels between AIN cases and non-AIN controls on the x-axis (log_2_ scale). Fold differences were calculated from NPX values as differences between values in cases versus controls. We calculated *Q* values using the Benjamini-Hochberg procedure for multiple comparisons. We also presented data using alternate methods for accounting for multiple comparisons using the Simes method and Bonferroni correction. In a sensitivity analysis, we only included proteins that were detectable in at least 75% of the samples. We used Ingenuity Pathway Analysis (IPA) content version 68752261 (release date: September 6, 2021) to identify potential top upstream regulators of the observed changes in urine protein expression.

For biomarker analyses, we presented characteristics of participants at biopsy as median (IQR) or count (percentage) by CXCL9 tertiles. We tested differences between groups using nonparametric trend tests (41). We showed correlation plots and correlation coefficients for CXCL9 measured by Olink and immunoassay. We tested the association of CXCL9 with adjudicated AIN using Wilcoxon rank-sum tests. In a supplementary analysis, we tested the association of CXCL9 using majority-adjudicated (rather than consensus) diagnoses, histological diagnoses, as reported on the official biopsy report, and the clinical nephrologists’ postbiopsy diagnoses. We also compared CXCL9 values between AIN and various control groups using the Kruskal-Wallis test. In our primary analysis, we indexed CXCL9 to urine creatinine to account for urine concentration differences affecting biomarker values, whereas in a supplementary analysis, we used unindexed values. We tested the association of various histological features with CXCL9 levels using nonparametric trend tests.

We tested the independent association of CXCL9 with AIN diagnosis using logistic regression analysis. We reported odds ratios (and 95% CIs) for AIN diagnosis per doubling of CXCL9 as well as for the 2 highest quartiles using the lowest quartile as the reference group. Model 1 investigated the univariable association of CXCL9 with AIN. To compare the additional value of the biomarkers over clinical information, we fit 2 additional models. Model 2 controlled for the clinical nephrologists’ prebiopsy suspicion of AIN (yes/no), obtained through chart review. Model 3 controlled for AIN diagnostic index, a recently developed model of 4 clinically available variables that was validated for histological AIN diagnosis (19). The degree of missingness of key covariates included in these models is noted in Supplemental Table 12. In a sensitivity analysis, we tested the association of CXCL9 with AIN after adjusting for demographics (age, sex, and race), comorbidities (diabetes and hypertension), plasma CXCL9 (log_2_ transformed), and urine IL-9 and TNF-α (log transformed). We reported the AUC for AIN diagnosis and its 95% CI for CXCL9, clinicians’ prebiopsy impressions, and the AIN diagnostic model. We then reported the increase in AUC when CXCL9 was added to the latter 2 models and compared models with and without CXCL9 using likelihood ratio tests.

In external validation cohorts, we presented characteristics of participants at biopsy as medians (IQRs) or counts (percentages) by site of enrollment. We compared CXCL9 levels between participants with AIN and controls using rank-sum tests and between AIN and various control subtypes using the Kruskal-Wallis test. We also presented AUCs of CXCL9 for AIN diagnosis. In a supplementary analysis, we presented biomarker comparisons by site. We presented CXCL9 test characteristics including sensitivity, specificity, and positive and negative predictive values in the discovery and validation cohorts at the 25^th^, 50^th^, and 75^th^ percentiles of CXCL9 values for the discovery cohort. We also presented test characteristics at an optimal cutoff derived using the Youden index, which maximizes the sum of sensitivity and specificity (42). We compared nanostring-derived expression values of *CXCL9* (transcript counts) between AIN and various control groups using rank-sum tests. We also presented the top differentially expressed genes between participants with AIN and individuals in the control group.

To determine an optimal combination of biomarkers, we used a feature selection algorithm called least absolute shrinkage and selection operator (LASSO), where we included all 16 biomarkers measured in this cohort. We used 1,000 iterations of randomly generated subsets consisting of 70% of the discovery cohort to determine the biomarkers most consistently associated with AIN diagnosis and included biomarkers selected in more than 75% of these models. We fit a logistic regression model of these biomarkers for the outcome of AIN in the first 70% of enrolled participants in the discovery cohort by enrollment date (training set) and applied model weights derived from this analysis to the last 30% of the discovery cohort by enrollment date (held-out test set) as well as external validation cohorts. We presented AUCs from the held-out test set and external validation cohort. We showed precision-recall curves and calibration plots. As the proportion of participants with AIN among those with AKI had been noted to be between 10% and 20%, we showed test characteristics at 2 AIN probability cutoffs (10% and 20%) with CXCL9 alone and with all 3 biomarkers. We used multiple imputations to account for missing data. We used Stata Statistical Software: Release 17.0 (StataCorp LP) for all analyses. All statistical tests were 2-sided with a significance level of 0.05.

### Study approval.

This study was approved by the Yale Human Investigation Committee (approval #11110009286). All participants provided written informed consent. The Icahn School of Medicine Mount Sinai validation cohort was approved by the IRB with approval number 14-00700. The C-PROBE of the George M. O’Brien Kidney Translational Core Center at the University of Michigan cohort was approved by the IRB with approval number HUM00178688.

### Data availability.

The biomarker and proteomics data set and the Dryad data set can be found in Moledina (43) and at https://datadryad.org/stash/dataset/doi:10.5061/dryad.ksn02v788

## Author contributions

DGM, RBC, LGC, JSP, FPW, and CRP designed the research studies. WO, RNS, and IR conducted the experiments. DGM, WO, GM, M Kashgarian, M Kuperman, KNC, SL, KM, MB, MAP, MES, and RLL acquired data. DGM and RNS analyzed data. DGM, LGC, FPW, and CRP wrote the manuscript. All authors approved final version.

## Supplementary Material

Supplemental data

Trial reporting checklists

ICMJE disclosure forms

## Figures and Tables

**Table 1:**
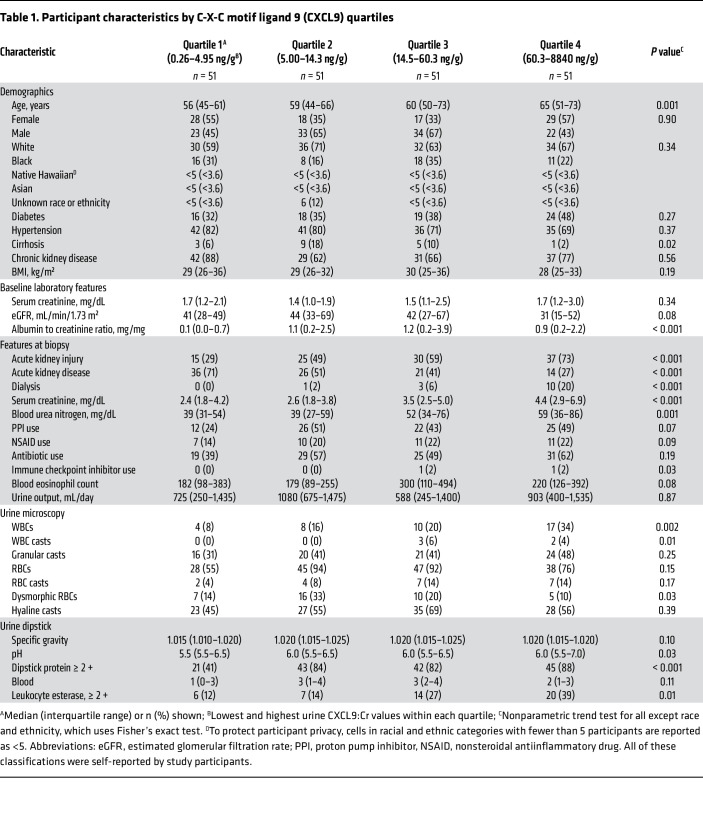
Participant characteristics by C-X-C motif ligand 9 (CXCL9) quartiles

**Table 2:**
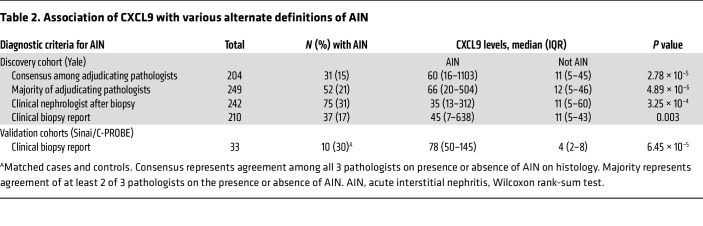
Association of CXCL9 with various alternate definitions of AIN

**Table 3:**
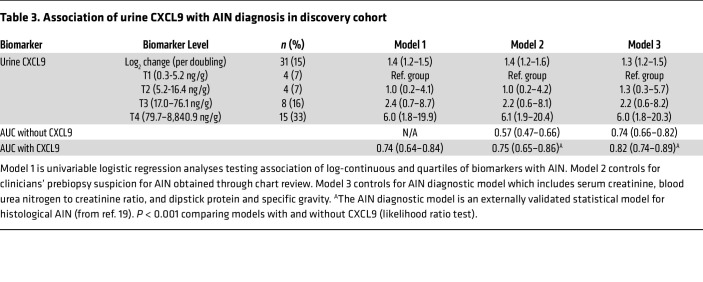
Association of urine CXCL9 with AIN diagnosis in discovery cohort

**Table 4:**
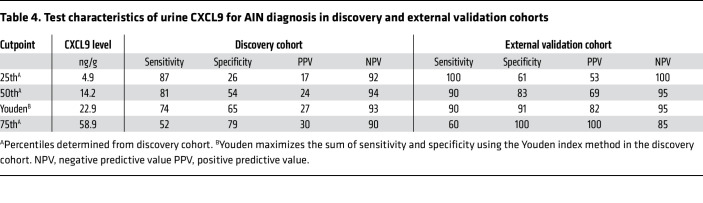
Test characteristics of urine CXCL9 for AIN diagnosis in discovery and external validation cohorts

## Version 1 07/03/2023

Electronic publication

## Version 2 03/15/2024

Updated with corrigendum
