# Identification and validation of urinary CXCL9 as a biomarker for diagnosis of acute interstitial nephritis

**DOI:** 10.1172/JCI180583

**Published:** 2024-03-15

**Authors:** Dennis G. Moledina, Wassim Obeid, Rex N. Smith, Ivy Rosales, Meghan E. Sise, Gilbert Moeckel, Michael Kashgarian, Michael Kuperman, Kirk N. Campbell, Sean Lefferts, Kristin Meliambro, Markus Bitzer, Mark A. Perazella, Randy L. Luciano, Jordan S. Pober, Lloyd G. Cantley, Robert B. Colvin, F. Perry Wilson, Chirag R. Parikh

Original citation: *J Clin Invest*. 2023;133(13):e168950. https://doi.org/10.1172/JCI168950

Citation for this corrigendum: *J Clin Invest*. 2024;134(6):e180583. https://doi.org/10.1172/JCI180583

In the abstract and the legend for Figure 1, the descriptions of the Olink Proteomics assays utilized were incorrect. The correct descriptions appear below. The HTML and PDF versions of the article have been updated online.

## Abstract

**Methods**. In a prospectively enrolled cohort with pathologist-adjudicated histological diagnoses, termed the discovery cohort, we tested the association of 180 immune proteins measured by a proximity extension assay with AIN and validated the top protein, CXCL9, using sandwich immunoassay.

**Results**. In a proximity extension assay, urinary CXCL9 was 7.6-fold higher in patients with AIN than in individuals in the control group (*P* = 1.23 × 10^–5^).

**Conclusion**. We identified CXCL9 as a diagnostic biomarker for AIN using proximity extension urine proteomics, confirmed this association using sandwich immunoassays in discovery and external validation cohorts, and observed higher expression of this protein in kidney biopsies from patients with AIN.

## Figure 1

Volcano plot demonstrating associations of proximity extension measurement of urine proteins with acute interstitial nephritis diagnosis.

The authors regret the errors. 

## Version 1 03/15/2024

Electronic publication

