# Supplementary material for: Identification and validation of urinary CXCL9 as a biomarker for diagnosis of acute interstitial nephritis
Source: J Clin Invest. 2023 Jul 3;133(13):e168950. doi: 10.1172/JCI168950 (PMC10313360; doi:10.1172/JCI168950)
Supplement: Supplemental data [file jci-133-168950-s010.pdf]

SUPPLEMENTAL FILE

Identification and validation of urine CXCL9 as a biomarker for diagnosis of acute interstitial nephritis

Moledina et al

Figure S1. Volcano plot showing urine proteins associated with AIN diagnosis after indexing to urine creatinine. 2

Figure S2. Volcano plot showing urine proteins associated with AIN diagnosis after excluding proteins detectable in <75% of samples. 3

Figure S3. STARD flow diagram 4

Figure S4. Correlation between urine CXCL9 measured by Olink proteomics and Mesoscale Discovery sandwich immunoassay 5

Figure S5. CXCL9 levels were higher in patients with AIN than in controls in the discovery cohorts without indexing to creatinine 6

Figure S6. CXCL9 levels were higher in patients with AIN than in controls with acute tubular injury. 7

Figure S7. Association of urine CXCL9 with severity of histological features. Non-parametric trend test; severity of interstitial features as determined by median value of three adjudicating pathologists. 8

Figure S8. Association of CXCL9 with glomerular crescents. Data on glomerular crescents obtained through review of official biopsy reports. 9

Figure S9. CXCL9 levels were higher in patients with AIN than in controls in each external validation cohort 10

Figure S10. Urine biomarker selection using LASSO feature selection algorithm 11

Figure S11. Receiver operating characteristics analysis of the 3-biomarker model in the test and external validation sets. 12

Figure S12. Precision recall curve 13

Figure S13. Calibration plots 14

Table S1. Histological diagnosis among participants included for urine proteomics analysis 15

Table S2. Baseline characteristics of participants in urine proteomic analysis 16

Table S3. Association of urine proteome with AIN diagnosis 17

Table S4. Pathway analysis demonstrating top upstream regulators of observed changes in urine proteome 18

Table S5. Independent association of urine CXCL9 with AIN diagnosis using alternative covariates 19

Table S6. Baseline characteristics of participants in external validation cohorts 20

Table S7. Top differentially-expressed genes in kidneys from patients with AIN and controls 21

Table S8. Test characteristics at various cutoffs 22

Table S9. List of proteins included in proteomics assay 23

Table S10. Quality control characteristics of Olink panels 24

Table S11. Assay characteristics of CXCL9 measured using Mesoscale discovery sandwich immunoassay25

Table S12. Missingness of key covariates 26

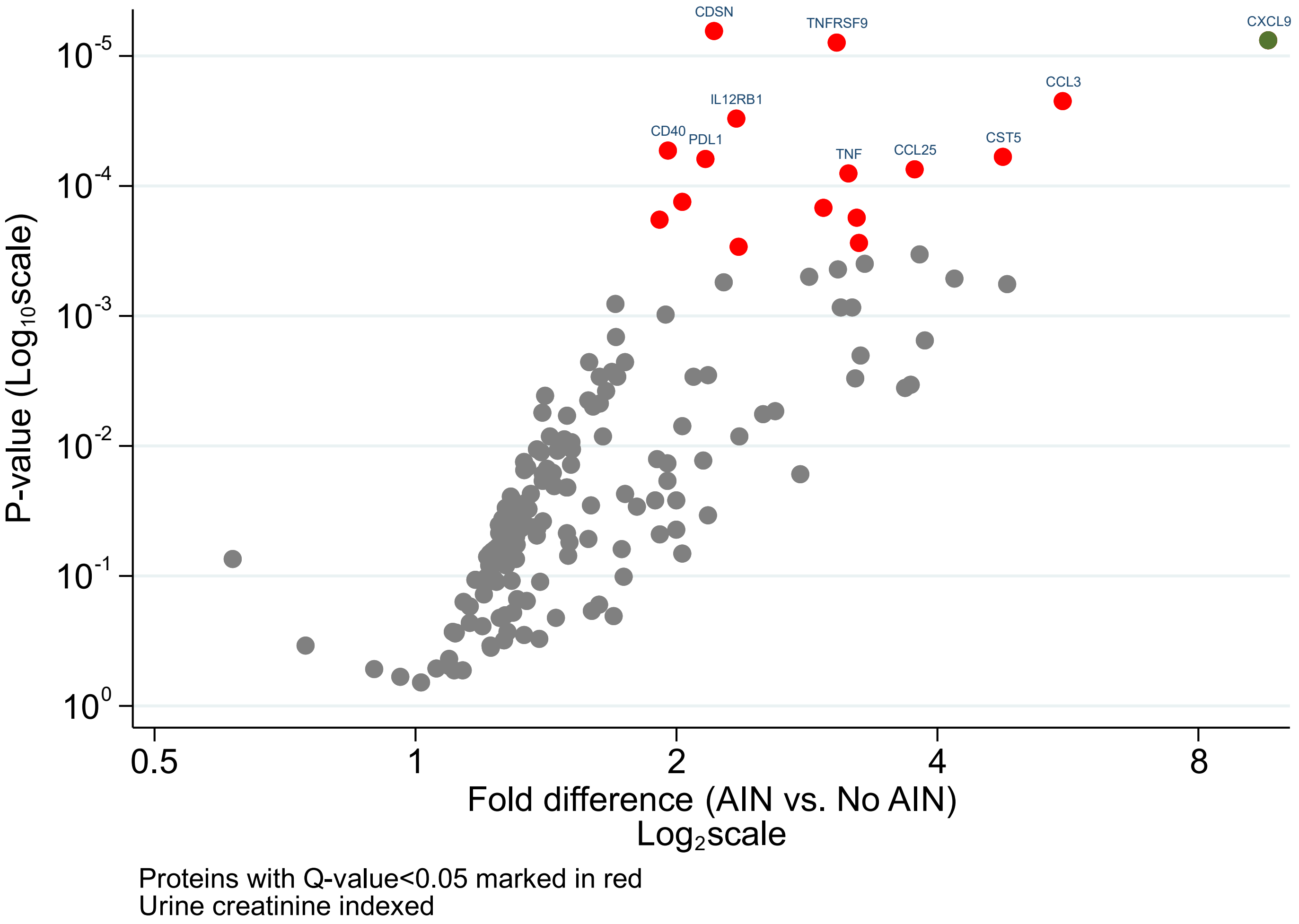

Figure S1. Volcano plot showing urine proteins associated with AIN diagnosis after indexing to urine creatinine.

Proteins with Q values <0.05 using the Benjamini-Hochberg procedure are highlighted in red. CXCL9 is highlighted in green.

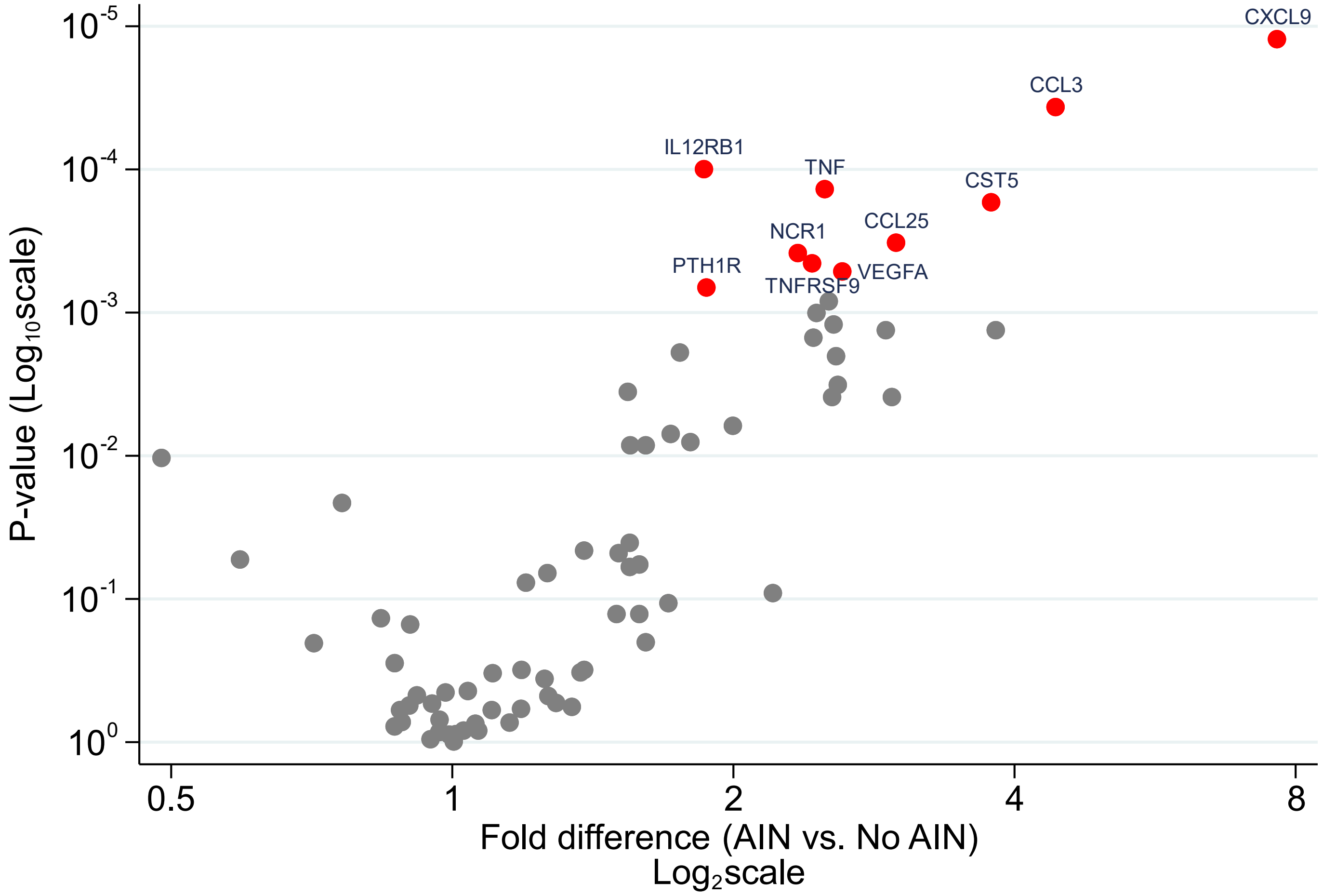

Figure S2. Volcano plot showing urine proteins associated with AIN diagnosis after excluding proteins detectable in <75% of samples.

Proteins with Q values <0.05 using the Benjamini-Hochberg procedure are highlighted in red.

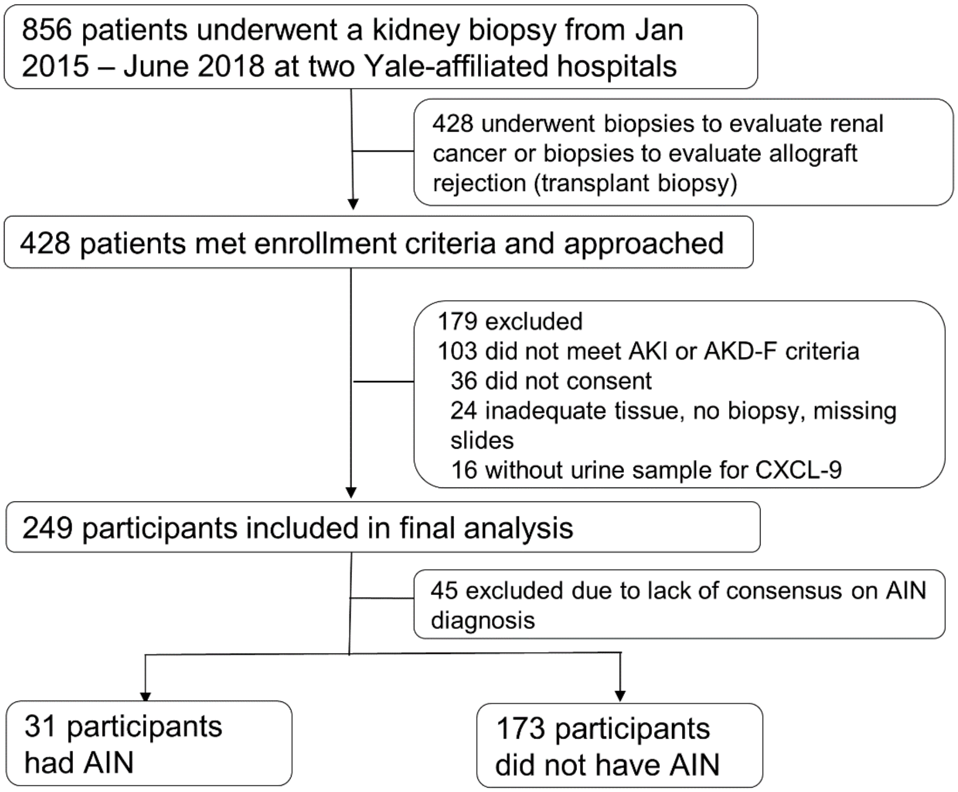

Figure S3. STARD flow diagram

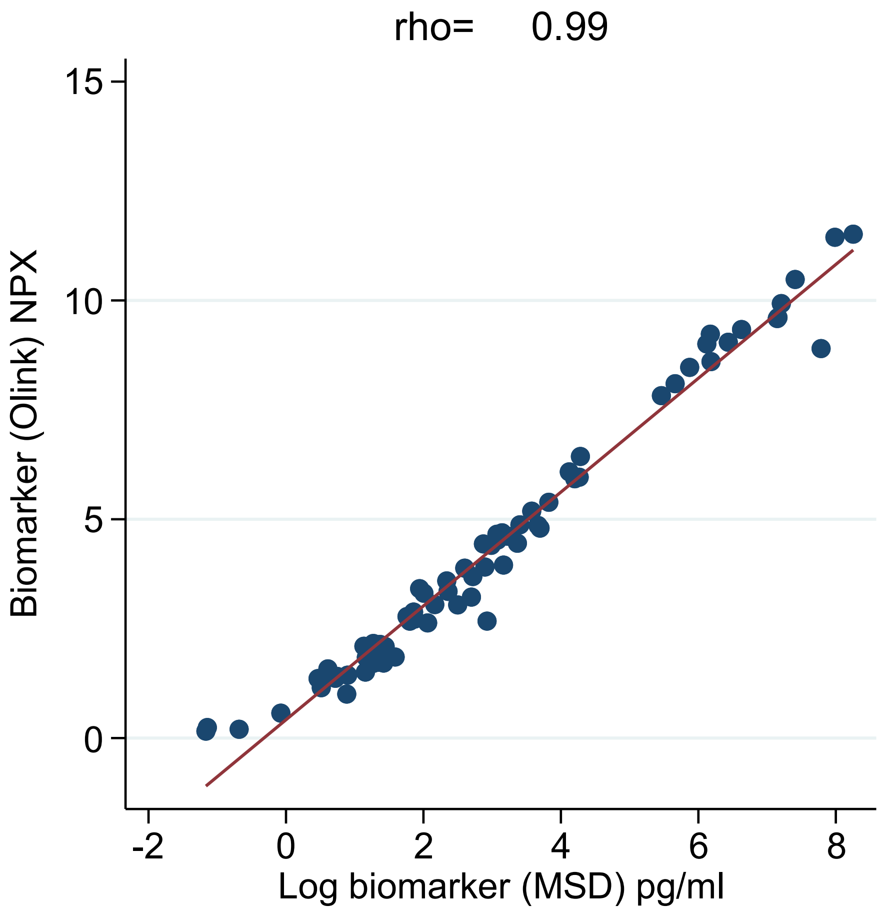

Figure S4. Correlation between urine CXCL9 measured by Olink proteomics and Mesoscale Discovery sandwich immunoassay

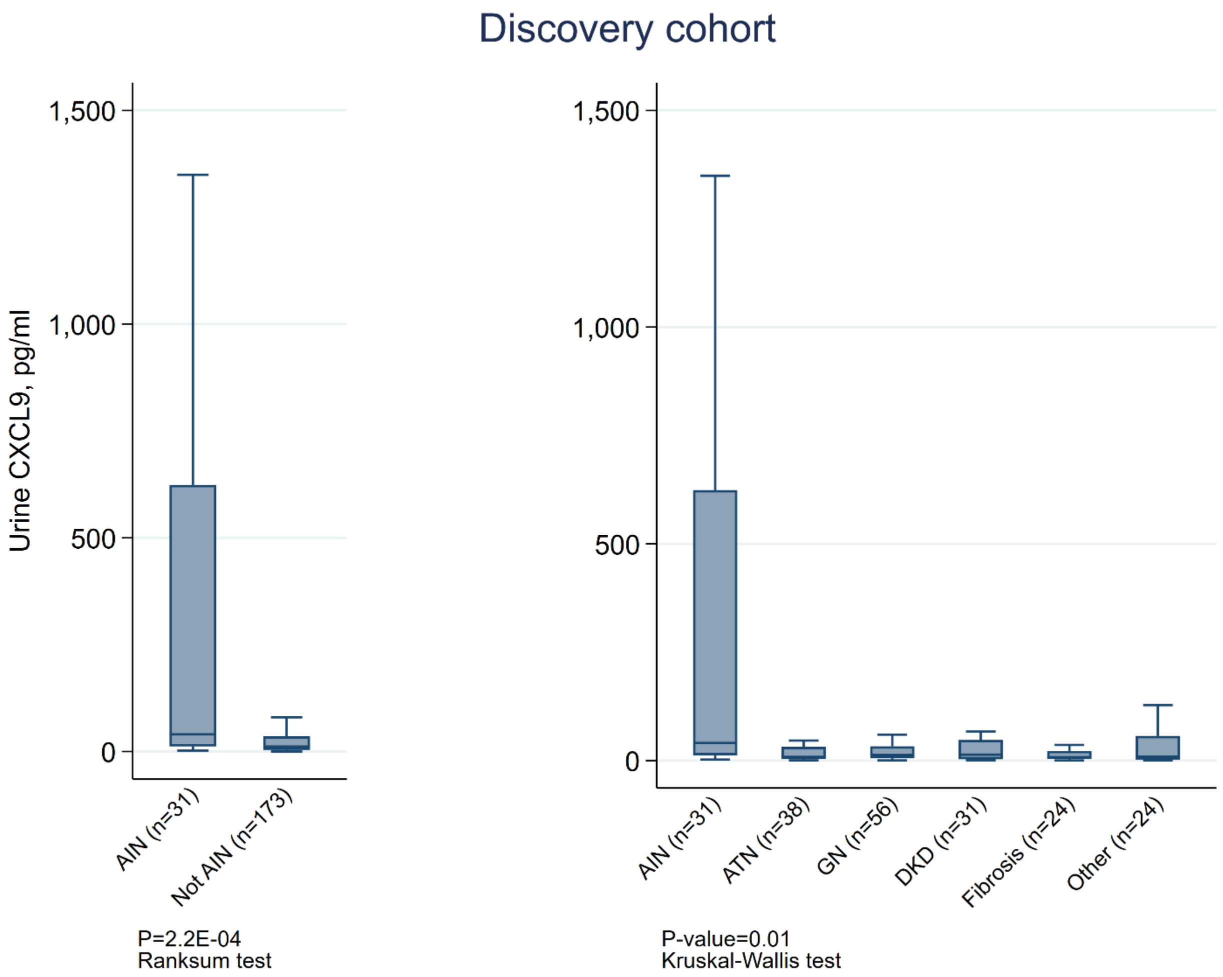

Figure S5. CXCL9 levels were higher in patients with AIN than in controls in the discovery cohorts without indexing to creatinine

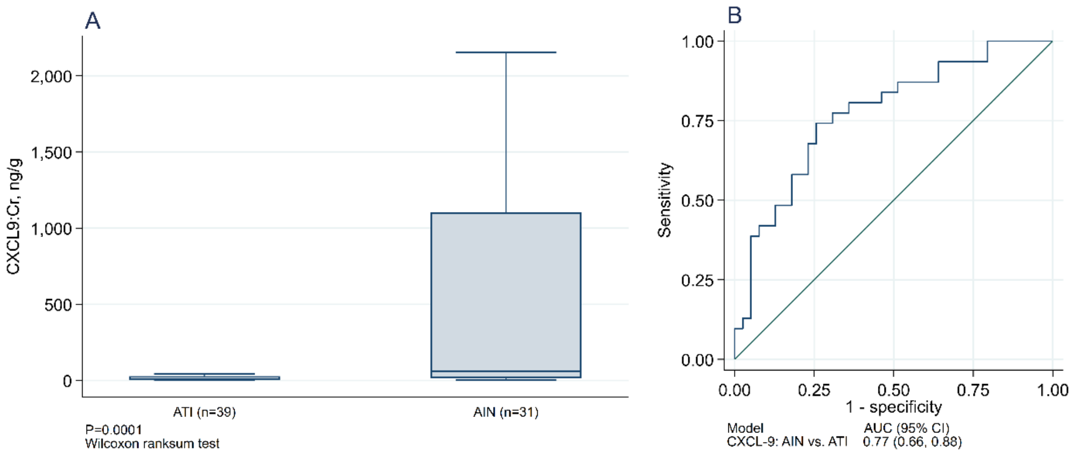

Figure S6. CXCL9 levels were higher in patients with AIN than in controls with acute tubular injury.

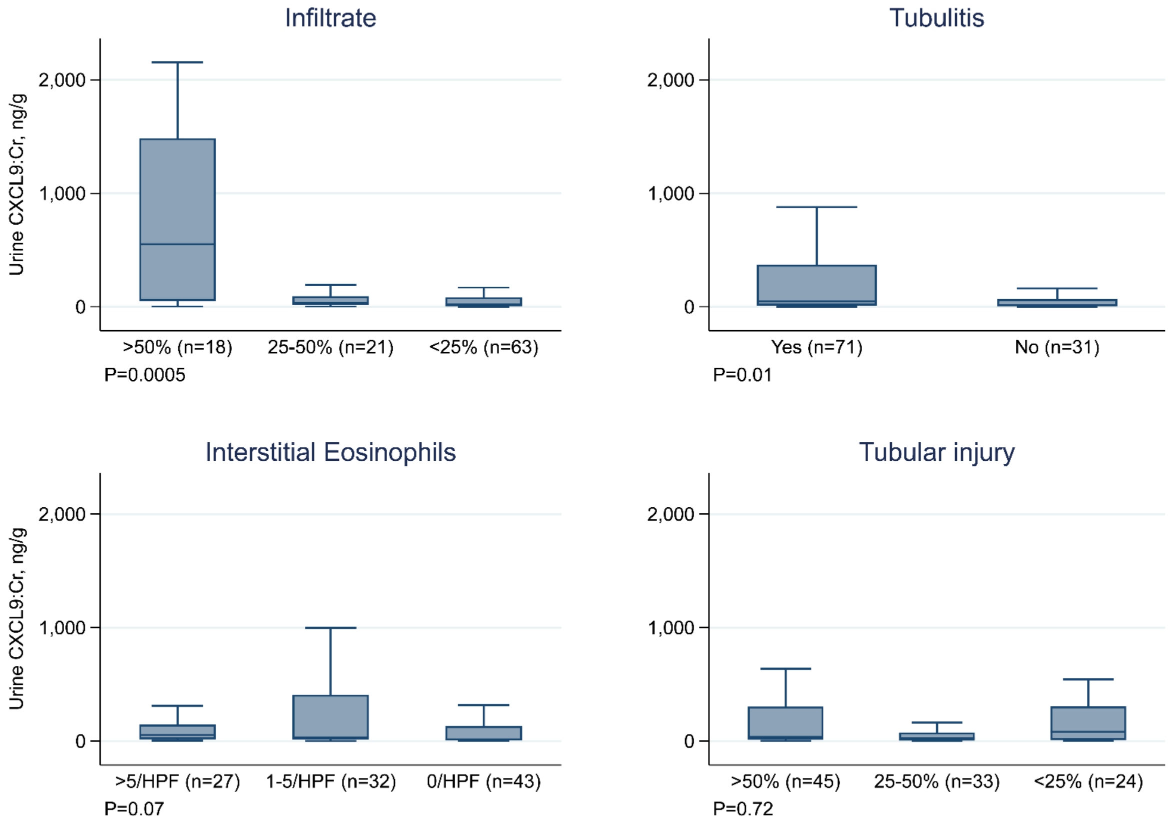

Figure S7. Association of urine CXCL9 with severity of histological features. Non-parametric trend test; severity of interstitial features as determined by median value of three adjudicating pathologists.

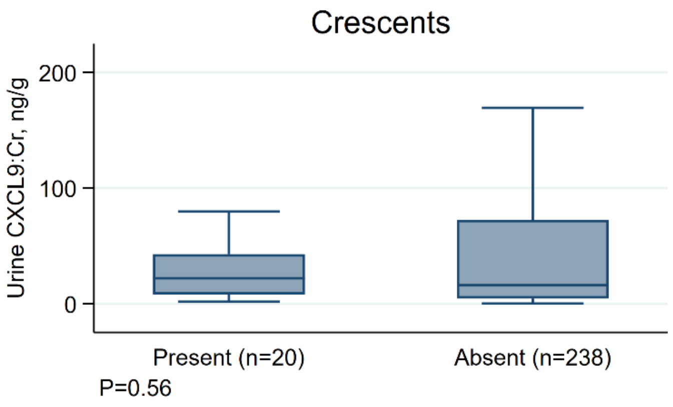

Figure S8. Association of CXCL9 with glomerular crescents. Data on glomerular crescents obtained through review of official biopsy reports.

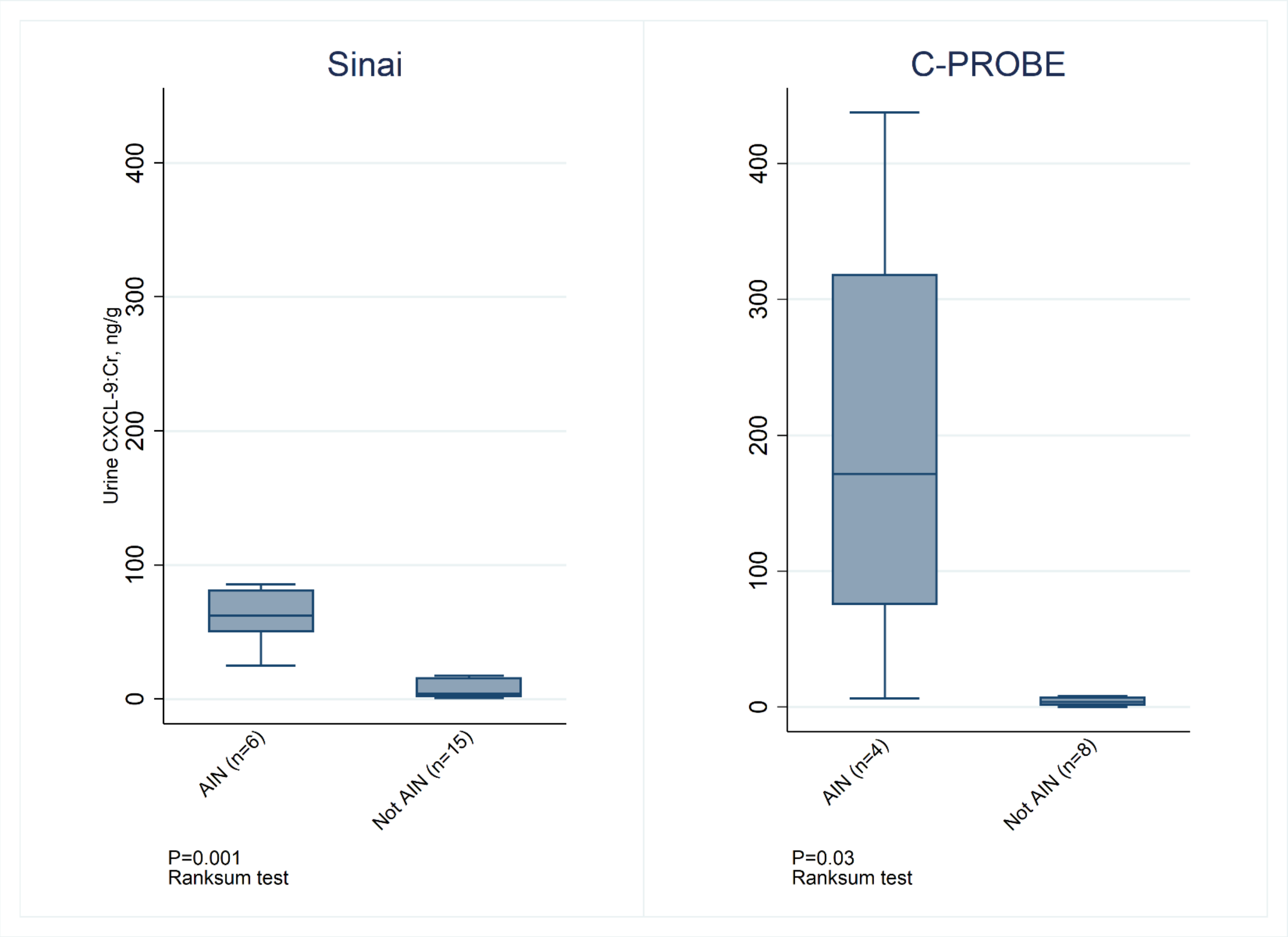

Figure S9. CXCL9 levels were higher in patients with AIN than in controls in each external validation cohort

Interaction *P* value = 0.83

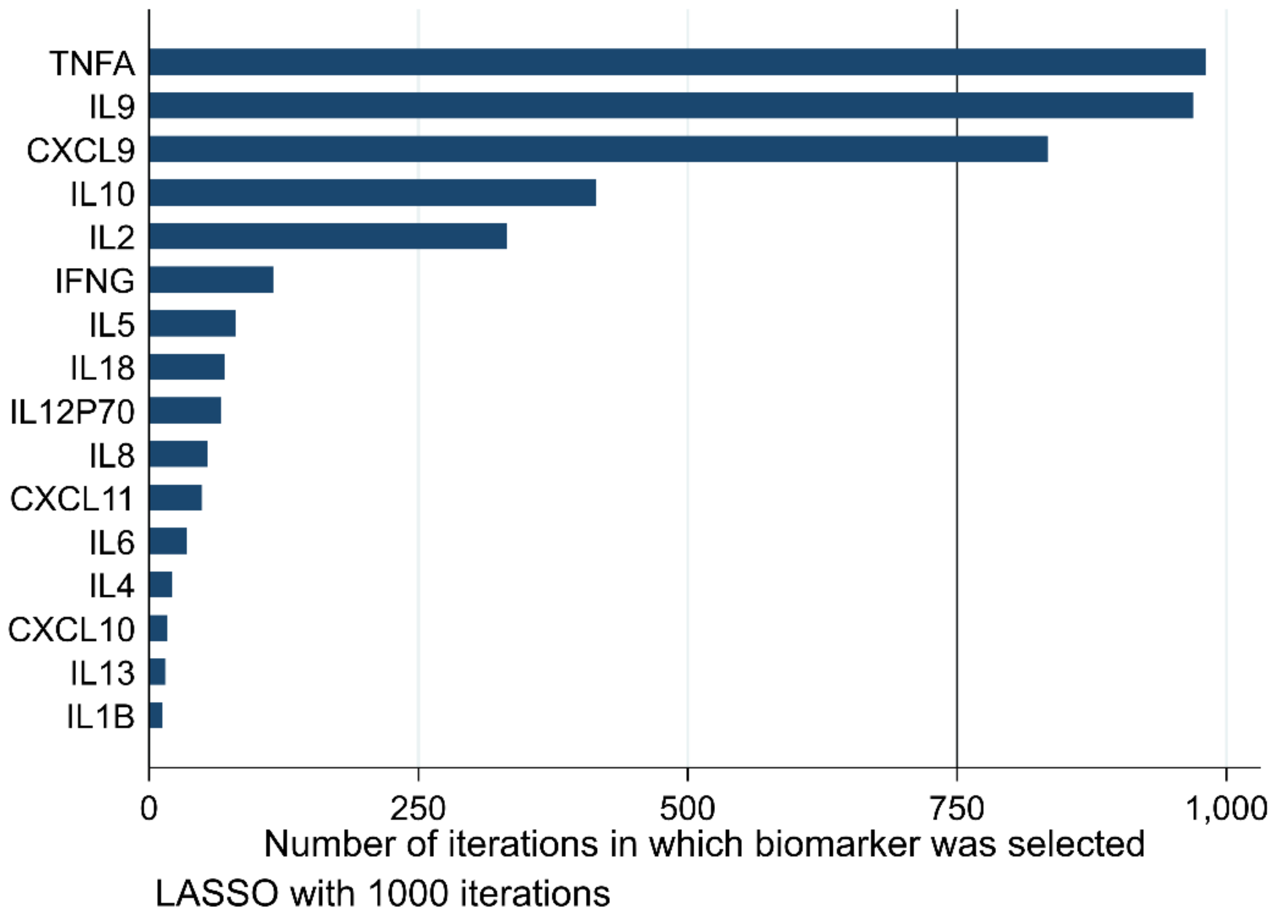

Figure S10. Urine biomarker selection using LASSO feature selection algorithm

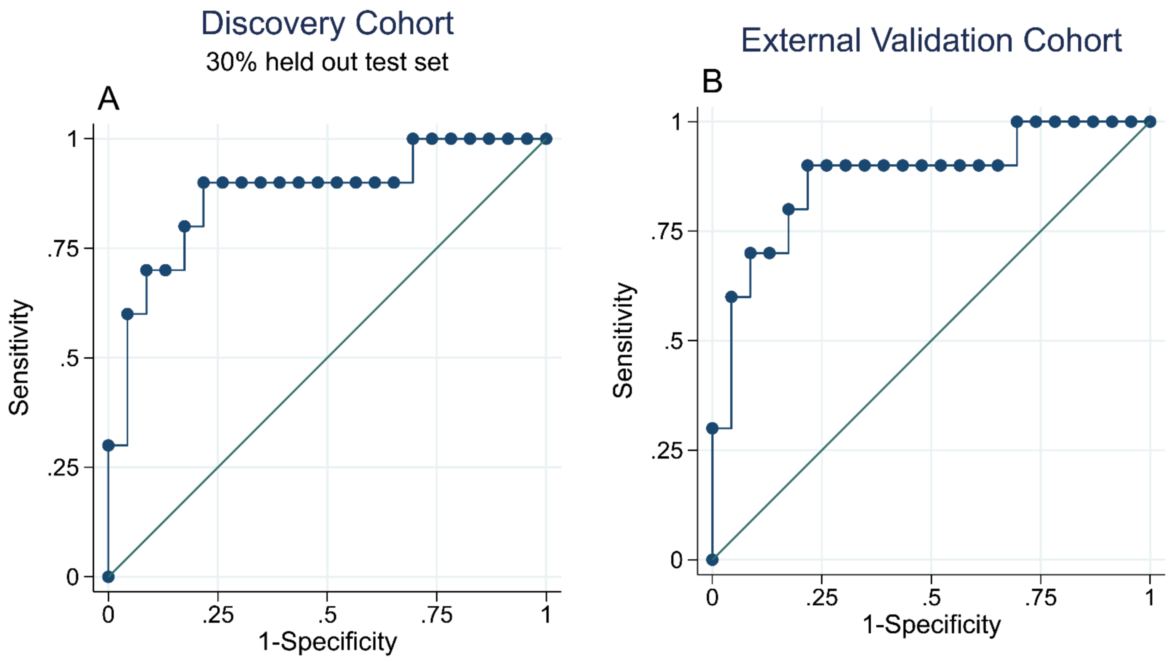

Figure S11. Receiver operating characteristics analysis of the 3-biomarker model in the test and external validation sets.

The 3 biomarker model is a logistic regression analysis for outcome of acute interstitial nephritis diagnosis derived in the discovery cohort: training set and model weights applied to the discovery cohort (held-out test set, Panel A) and the external validation cohort (Panel B).

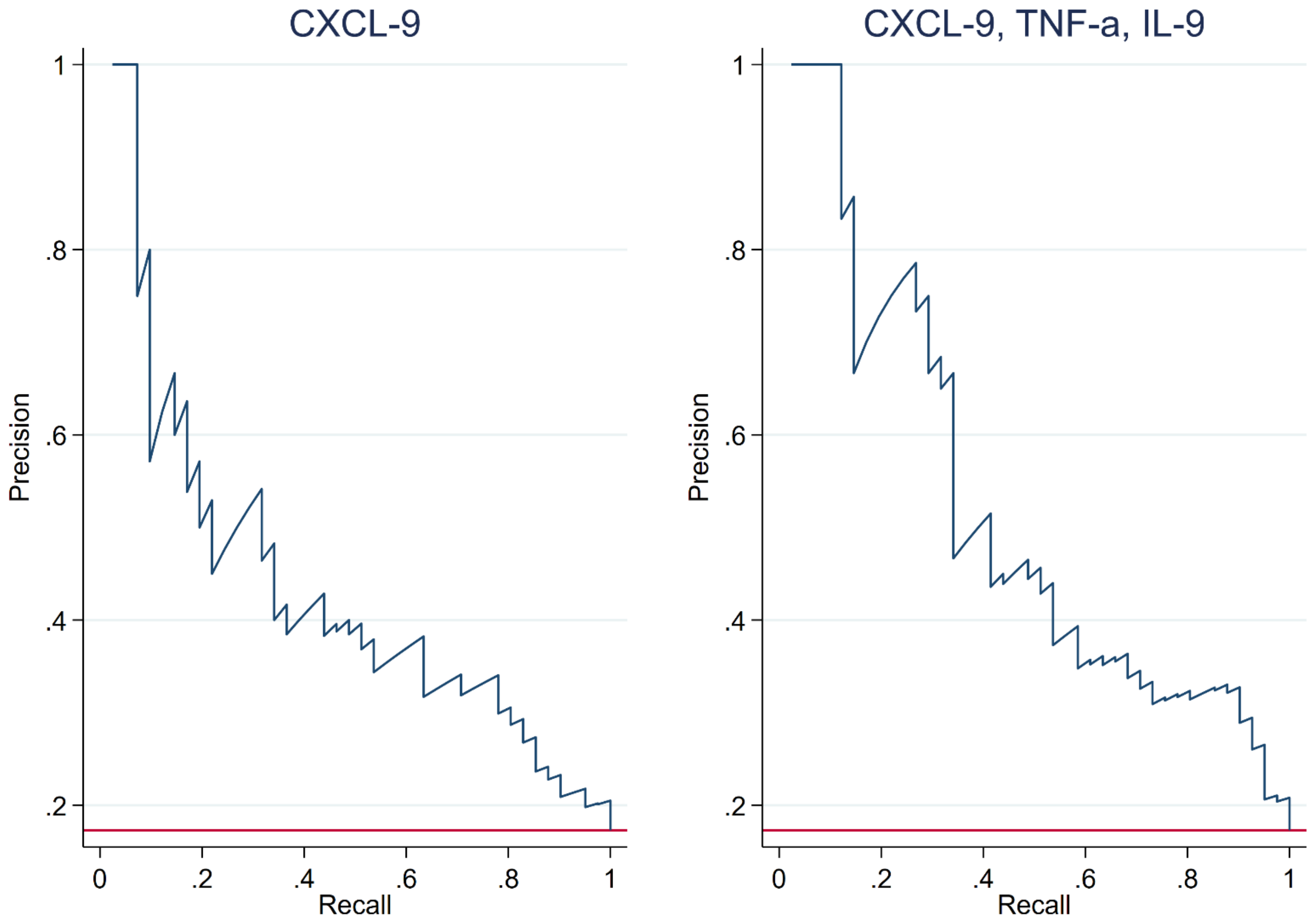

Figure S12. Precision recall curve

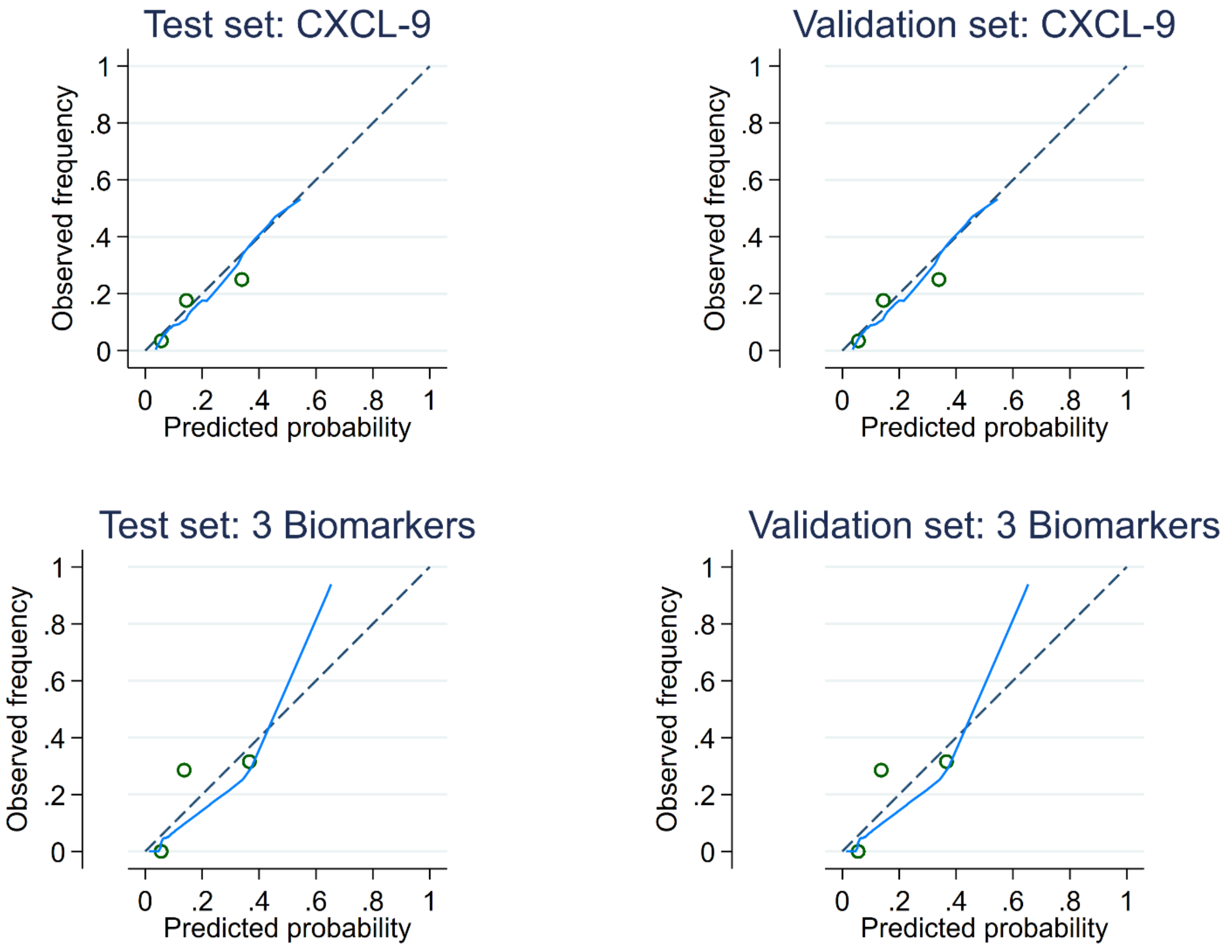

Figure S13. Calibration plots

Table S1. Histological diagnosis among participants included for urine proteomics analysis

Diagnosis	n	Classification
Acute interstitial Nephritis	31	AIN case
Acute tubular injury	9	Non-AIN control
Diabetic kidney disease	10	Non-AIN control
Glomerular disease	23	Non-AIN control
Arterionephrosclerosis	15	Non-AIN control

Table S2. Baseline characteristics of participants in urine proteomic analysis

Characteristic	Not AIN (n = 57)	AIN (n =3 1)	*P* Value
**Demographics**			
Age, y	56 (47-64)	58 (39-67)	0.99
Female	30 (53)	17 (55)	0.84
Black race	16 (28)	10 (32)	0.68
**Comorbidities**			
Chronic kidney disease	34 (62)	20 (69)	0.52
Diabetes	23 (41)	7 (23)	0.08
Hypertension	44 (77)	20 (65)	0.20
Cirrhosis	4 (7)	0 (0)	0.13
**Baseline features**			
Baseline estimated glomerular filtration rate	46 (30-79)	29 (17-65)	0.06
Baseline creatinine, mg/dL	1.2 (1.0- 2.0)	1.9 (1.0-3.1)	0.10
**Features at biopsy**			
Acute kidney injury	16 (38)	18 (58)	0.09
Acute kidney disease	26 (62)	13 (42)	0.09
Hospitalized	23 (40)	24 (77)	0.001
Creatinine at biopsy	2.9 (1.7-4.7)	4.6 (3.2- 6.3)	0.002
Baseline albumin to creatinine ratio, mg/mg	0.9 (0.1-2.6)	0.1 (0.0-1.7)	0.02

Median (interquartile range) or n (%) shown; Chi^2 or Kruskal Wallis test; values <0.05 bolded.

Abbreviations: AIN, acute interstitial nephritis

Table S3. Association of urine proteome with AIN diagnosis

Protein	UniProt ID	*P* Value	*Q* Value (Simes)	*Q* Value (B-H)	Bonferroni *P* Value	Fold Difference
**Significantly different proteins**						
**CXCL9**	Q07325	1.23E-05	2.22E-03	2.22E-03	2.22E-03	7.6
**CCL3**	P10147	3.67E-05	3.30E-03	6.57E-03	6.60E-03	4.4
**FGF19**	O95750	1.07E-04	4.82E-03	1.89E-02	1.93E-02	3.0
**IL12RB1**	P42701	9.96E-05	4.82E-03	1.77E-02	1.79E-02	1.9
**TNF**	P01375	1.37E-04	4.94E-03	2.42E-02	2.47E-02	2.5
**CST5**	P28325	1.70E-04	5.09E-03	2.97E-02	3.05E-02	3.8
**IL15RA**	Q13261	2.31E-04	5.95E-03	4.03E-02	4.17E-02	1.5
**Additional relevant proteins**						
**CXCL10**	P02778	9.10E-02	1.93E-01	9.90E-01	1.00E+00	2.2
**CXCL11**	O14625	4.32E-02	1.18E-01	9.90E-01	1.00E+00	2.1

AIN vs. No AIN

Table S4. Pathway analysis demonstrating top upstream regulators of observed changes in urine proteome

Upstream Regulator	Predicted Activation State	Activation z-score	*P* value of overlap	B-H corrected *P* value
**IL10**	Inhibited	-3.341	5.04E-34	2.13E-30
**IFNG**	Activated	4.586	3.89E-29	8.21E-26
**IL12 (complex)**	Activated	3.013	6E-29	8.45E-26
**RNASE2**	Activated	3.786	2.85E-28	3.01E-25
**IL4**	Activated	3.369	9.14E-28	7.72E-25
**Immunoglobulin**	Activated	2.585	7.55E-27	5.31E-24
**NFkB (complex)**	Activated	4.882	4.46E-25	2.69E-22
**RNASE1**	Activated	3.44	8.81E-25	4.65E-22
**IL18**	Activated	4.072	2.71E-24	1.27E-21
**IL1B**	Activated	4.981	4.09E-24	1.73E-21

Table S5. Independent association of urine CXCL9 with AIN diagnosis using alternative covariates

Model	Adjusted odds ratio (95% CI)
**Univariable**	1.35 (1.19-1.54)
**Demographics***	1.44 (1.24-1.67)
**Age**	1.44 (1.25-1.67)
**Comorbidities**	1.40 (1.22-1.62)
**Plasma CXCL9**	1.39 (1.17-1.65)
**Urine Interleukin-9**	1.23 (1.06-1.43)
**Urine TNF-a**	1.20 (1.03-1.41)
**CXCL10**	1.39 (1.14, 1.70)

*Abbreviations: TNF, tumor necrosis factor; *controls for age, sex, black race*

Table S6. Baseline characteristics of participants in external validation cohorts

Characteristic	Sinai (n = 21)	CPROBE (n = 12)
**Demographics**		
Age, y	57 (45-63)	59.9 (50, 74.5)
Female	13 (61.9)	3 (25)
Black race	4 (19)	1 (8)
**Comorbidities**		
Chronic kidney disease	13 (61.9)	12 (100)
Diabetes	6 (28.6)	3 (25)
Hypertension	13 (61.9)	9 (75)
Cirrhosis	1 (4.8)	0 (0)
**Baseline features**		
Baseline estimated glomerular filtration rate	35.93 (25.80-52.51)	50.58 (28.06, 58.87)
Baseline Creatinine, mg/dl	1.56 (1.31-2.05)	1.2
**Features at biopsy**		
Acute kidney injury	1 (4.8)	2 (17)
Acute kidney disease	3 (14.3)	N/A
Hospitalized	2 (9.5)	N/A
Creatinine at biopsy	1.99 (1.54-2.35)	2.4 (1.6 2.8)
**Histological diagnosis**		
Acute interstitial Nephritis	6 (28.6)	4 (33)
Acute tubular injury	5 (23.7)	0
Diabetic kidney disease	4 (19)	0
Glomerular disease	0 (0%)	6 (50)
Arterionephrosclerosis	6 (28.6)	2 (17)

Values are median (IQR) or n (%)

Table S7. Top differentially-expressed genes in kidneys from patients with AIN and controls

Transcript	*Name*	*AIN vs All Others*	*IFN-ɣ–Induced*
IFI30	Gamma-interferon-inducible lysosomal thiol reductase	*3.49E-10*	*Yes (PMID: 32103982)*
HLA-B	Human leucocyte antigen-B (major histocompatibility complex, class I, B)	*2.40E-09*	*Yes (PMID:* 3084532*)*
ITGB2	Integrin beta chain-2 (CD18)	*1.24E-08*	
CD74	HLA-DR antigens-associated invariant chain; HLA class II histocompatibility antigen gamma chain	*1.26E-08*	*Yes (*PMID: 26039541*)*
LILRB4	Leukocyte immunoglobulin-like receptor subfamily B member 4	*1.34E-08*	
PDCD1LG2	Programmed cell death 1 ligand 2	*2.07E-08*	Yes (PMID: 28494868)
TLR8	Toll-like receptor 8	*3.06E-08*	*Yes (PMID: 28982143)*
CXCR3	Receptor for CXCL9	*3.71E-08*	*Yes (PMID: 29950350)*
HLA-DMB	HLA class II histocompatibility antigen, DM beta chain	*4.18E-08*	*Yes (PMID: 9144496)*
ITGAX	Integrin, alpha X (complement component 3 receptor 4 subunit)	*5.22E-08*	*Yes (PMID: 28982143)*

Table S8. Test characteristics at various cutoffs

	**CXCL9**		**CXCL9, TNF-a, IL-9**	
	Sensitivity	Specificity	Sensitivity	Specificity
**Test set**				
10%	81%	51%	87%	60%
20%	48%	82%	58%	77%
**External validation set**				
10%	90%	83%	90%	70%
20%	50%	100%	60%	91%

Table S9. List of proteins included in proteomics assay

Assay	UniProt ID	Assay	UniProt ID	Assay	UniProt ID	Assay	UniProt ID
ADA	P00813	DGKZ	Q13574	IL33	O95760	PTH1R	Q03431
AREG	P15514	DNER	Q8NFT8	IL4	P05112	SCF	P21583
ARNT	P27540	DPP10	Q8N608	IL6	P05231	SH2B3	Q9UQQ2
ARTN	Q5T4W7	DX	P51671	IL7	P13232	SH2D1A	O60880
AXIN1	O15169	EBP1	Q13541	IL8	P10145	SIRT2	Q8IXJ6
BACH1	O14867	EDAR	Q9UNE0	IRAK1	P51617	SIT1	Q9Y3P8
BETANGF	P01138	EGLN1	Q9GZT9	IRAK4	Q9NWZ3	SLAMF1	Q13291
BIRC2	Q13490	EIF4G1	Q04637	IRF9	Q00978	SPRY2	O43597
BTN3A2	P78410	EIF5A	P63241	ITGA11	Q9UKX5	SRPK2	P78362
CASP8	Q14790	ENRAGE	P80511	ITGA6	P23229	ST1A1	P50225
CCL19	Q99731	FAM3B	P58499	ITGB6	P18564	STAMBP	O95630
CCL20	P78556	FCRL3	Q96P31	ITM2A	O43736	STC1	P52823
CCL23	P55773	FCRL6	Q6DN72	JUN	P05412	TANK	Q92844
CCL25	O15444	FGF19	O95750	KLRD1	Q13241	TGFALPHA	P01135
CCL28	Q9NRJ3	FGF2	P09038	KPNA1	P52294	TNF	P01375
CCL3	P10147	FGF21	Q9NSA1	KRT19	P08727	TNFB	P01374
CCL4	P13236	FGF23	Q9GZV9	LAG3	P18627	TNFRSF9	Q07011
CD244	Q9BZW8	FGF5	P12034	LAMP3	Q9UQV4	TNFSF14	O43557
CD28	P10747	FLT3L	P49771	LAPTGFBETA1	P01137	TPSAB1	Q15661
CD40	P25942	FXYD5	Q96DB9	LIF	P15018	TRAF2	Q12933
CD5	P06127	FZ	P05113	LIFR	P42702	TRAIL	P50591
CD6	P30203	GALNT3	Q14435	LILRB4	Q8NHJ6	TRANCE	O14788
CD83	Q01151	GDNF	P39905	LY75	O60449	TREM1	Q9NP99
CD8A	P01732	GLB1	P16278	MASP1	P48740	TRIM21	P19474
CDCP1	Q9H5V8	HCLS1	P14317	MCP1	P13500	TRIM5	Q9C035
CDSN	Q15517	HEXIM1	O94992	MCP2	P80075	TSLP	Q969D9
CKAP4	Q07065	HGF	P14210	MCP3	P80098	TWEAK	O43508
CLEC4A	Q9UMR7	HNMT	P50135	MCP4	Q99616	UPA	P00749
CLEC4C	Q8WTT0	HSD11B1	P28845	MGMT	P16455	VEGFA	P15692
CLEC4D	Q8WXI8	ICA1	Q05084	MILR1	Q7Z6M3	ZBTB16	Q05516
CLEC4G	Q6UXB4	IFNGAMMA	P01579	MMP1	P03956		
CLEC6A	Q6EIG7	IFNLR1	Q8IU57	MMP10	P09238		
CLEC7A	Q9BXN2	IL10	P22301	NCR1	O76036		
CNTNAP2	Q9UHC6	IL10RA	Q13651	NF2	P35240		
CSF1	P09603	IL10RB	Q08334	NFATC3	Q12968		
CST5	P28325	IL12B	P29460	NRTN	Q99748		
CX3CL1	P78423	IL12RB1	P42701	NT3	P20783		
CXADR	P78310	IL13	P35225	NTF4	P34130		
CXCL1	P09341	IL15RA	Q13261	OPG	O00300		
CXCL10	P02778	IL17A	Q16552	OSM	P13725		
CXCL11	O14625	IL17C	Q9P0M4	PADI2	Q9Y2J8		
CXCL12	P48061	IL18	Q14116	PDL1	Q9NZQ7		
CXCL5	P42830	IL18R1	Q13478	PIK3AP1	Q6ZUJ8		
CXCL6	P80162	IL1ALPHA	P01583	PLXNA4	Q9HCM2		
CXCL9	Q07325	IL2	P60568	PPP1R9B	Q96SB3		
DAPP1	Q9UN19	IL20	Q9NYY1	PRDX1	Q06830		
DCBLD2	Q96PD2	IL20RA	Q9UHF4	PRDX3	P30048		
DCTN1	Q14203	IL22RA1	Q8N6P7	PRDX5	P30044		
DDX58	O95786	IL24	Q13007	PRKCQ	Q04759		
DFFA	O00273	IL2RB	P14784	PSIP1	O75475		

Table S10. Quality control characteristics of Olink panels

Characteristic	Value
Samples that passed quality control, n (%)	87 (99)
Average intra-assay coefficient of variation	
Immune response panel	5%
Inflammation panel	3%
Proteins detected in >75% of the samples	
Immune response panel	30/92
Inflammation panel	45/92

Table S11. Assay characteristics of CXCL9 measured using Mesoscale discovery sandwich immunoassay

Characteristic	Value
**Median (IQR) pg/ml**	13.76 (4.14-49.24)
**Detectable (%)**	99%
**LLOD (pg/mL)**	0.24
**ULOD (pg/mL)**	8000
**Inter-assay CV**	11%
**Intra-assay CV**	2%

N = 258 (31 with AIN); CV, coefficient of variation LLOD, lower limit of detection; ULOD, upper limit of detection

Table S12. Missingness of key covariates

Variable	Discovery (Yale)	Validation (Sinai/C-PROBE)
**Histological diagnosis**	0	0
**Urine CXCL9**	0	0
**Urine specific gravity**	0	26
**Urine dipstick protein**	0	0
**Serum creatinine**	0	6
**Urine protein to creatinine ratio**	0	0
